# Author Correction: Precise control of SCRaMbLE in synthetic haploid and diploid yeast

**DOI:** 10.1038/s41467-019-08474-w

**Published:** 2019-02-14

**Authors:** Bin Jia, Yi Wu, Bing-Zhi Li, Leslie A. Mitchell, Hong Liu, Shuo Pan, Juan Wang, Hao-Ran Zhang, Nan Jia, Bo Li, Michael Shen, Ze-Xiong Xie, Duo Liu, Ying-Xiu Cao, Xia Li, Xiao Zhou, Hao Qi, Jef D. Boeke, Ying-Jin Yuan

**Affiliations:** 1 Key Laboratory of Systems Bioengineering (Ministry of Education), School of Chemical Engineering and Technology, Tianjin University, Tianjin, 300072 China; 2 SynBio Research Platform, Collaborative Innovation Center of Chemical Science and Engineering, Tianjin, 300072 China; 3 Institute for Systems Genetics, New York University Langone Medical Center, 550 First Avenue, New York, NY 10016 USA

Correction to: *Nature Communications* 10.1038/s41467-018-03084-4; published online 22 May 2018

The original version of this Article omitted a declaration from the Competing Interests statement, which should have included the following: ‘J.D.B. is a founder and Director of the following: Neochromosome, Inc., the Center of Excellence for Engineering Biology, and CDI Labs, Inc. and serves on the Scientific Advisory Board of the following: Modern Meadow, Inc., Recombinetics, Inc., and Sample6, Inc.’. This has now been corrected in both the PDF and HTML versions of the Article.

