# Supplementary material for: Precise control of SCRaMbLE in synthetic haploid and diploid yeast
Source: Nat Commun. 2018 May 22;9:1933. doi: 10.1038/s41467-018-03084-4 (PMC5964104; doi:10.1038/s41467-018-03084-4)
Supplement: Supplementary file 1 — Supplementary Information [file 41467_2018_3084_MOESM1_ESM.pdf]

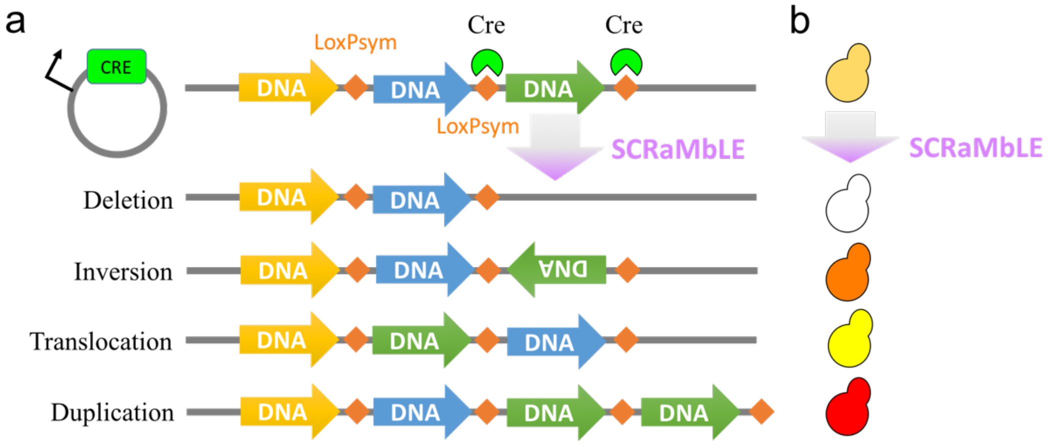

Supplementary Figure 1. SCRaMbLE leads to diversity generating desired phenotypes. (a) LoxPsym sites (orange diamonds) are inserted in the 3'UTR of each non-essential gene. The symmetry of loxPsym sites result in deletion, inversion, translocation and duplication, simultaneously. (b) Induction of SCRaMbLE in a synthetic strain results in a significant increase in diversity, generating desired phenotypes. SCRaMbLE events can be analyzed by deep sequencing and long-read sequencing.

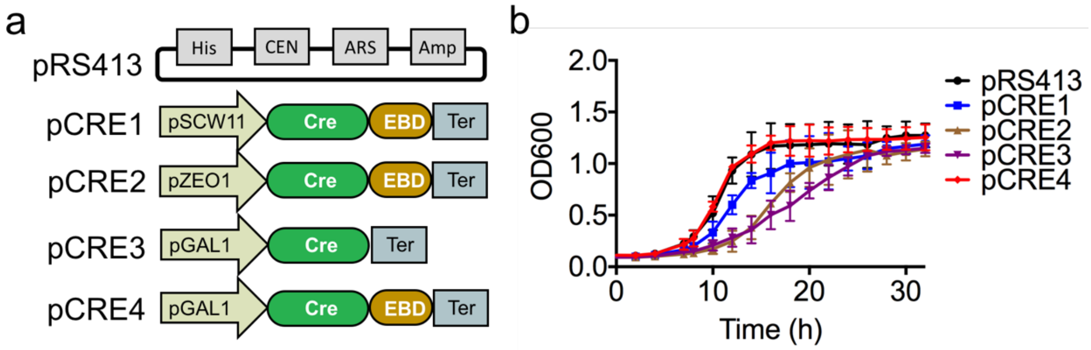

Supplementary Figure 2. Design of Cre switches for tightly regulation of Cre activity. (a) Design of different CRE switches and fitness assays in the synV yeast; pSCW11 is a daughter-cell-specific promoter； pZEO1 is a weak constitutive yeast promoter; The pGAL1 promoter is galactose inducible promoter; The Cre-EBD is a fusion protein of Cre recombinase and estrogen binding domain (EBD); The Ter is the CYC1 transcription terminator. (b) Growth curve of synV strains containing pCRE1, pCRE2, pCRE3, pCRE4 and pRS413, respectively. Growth of strains was assessed at 30℃ on SC-His medium for 32 hours. Error bars represent s.d. from three independent experiments.

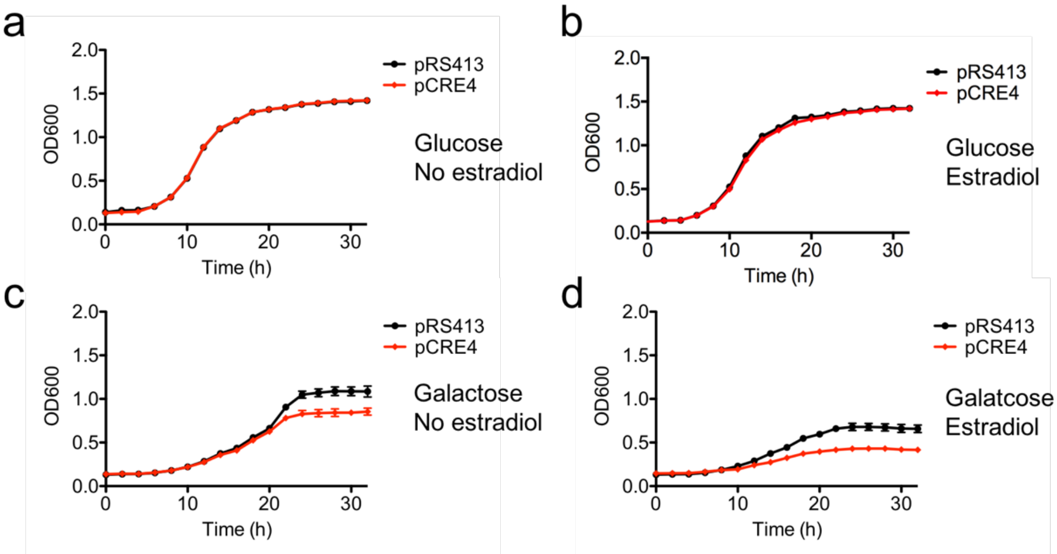

Supplementary Figure 3. Growth curve of synthetic strains contained pRS413 (control) and pCRE4 at different medium. (**a**) SC-His glucose medium without estradiol; (**b**) SC-His glucose medium with 1 μM estradiol; (**c**) SGal-His medium without estradiol; (**d**) SGal-His medium with 1 μM estradiol. Error bars represent s.d. from three independent experiments.

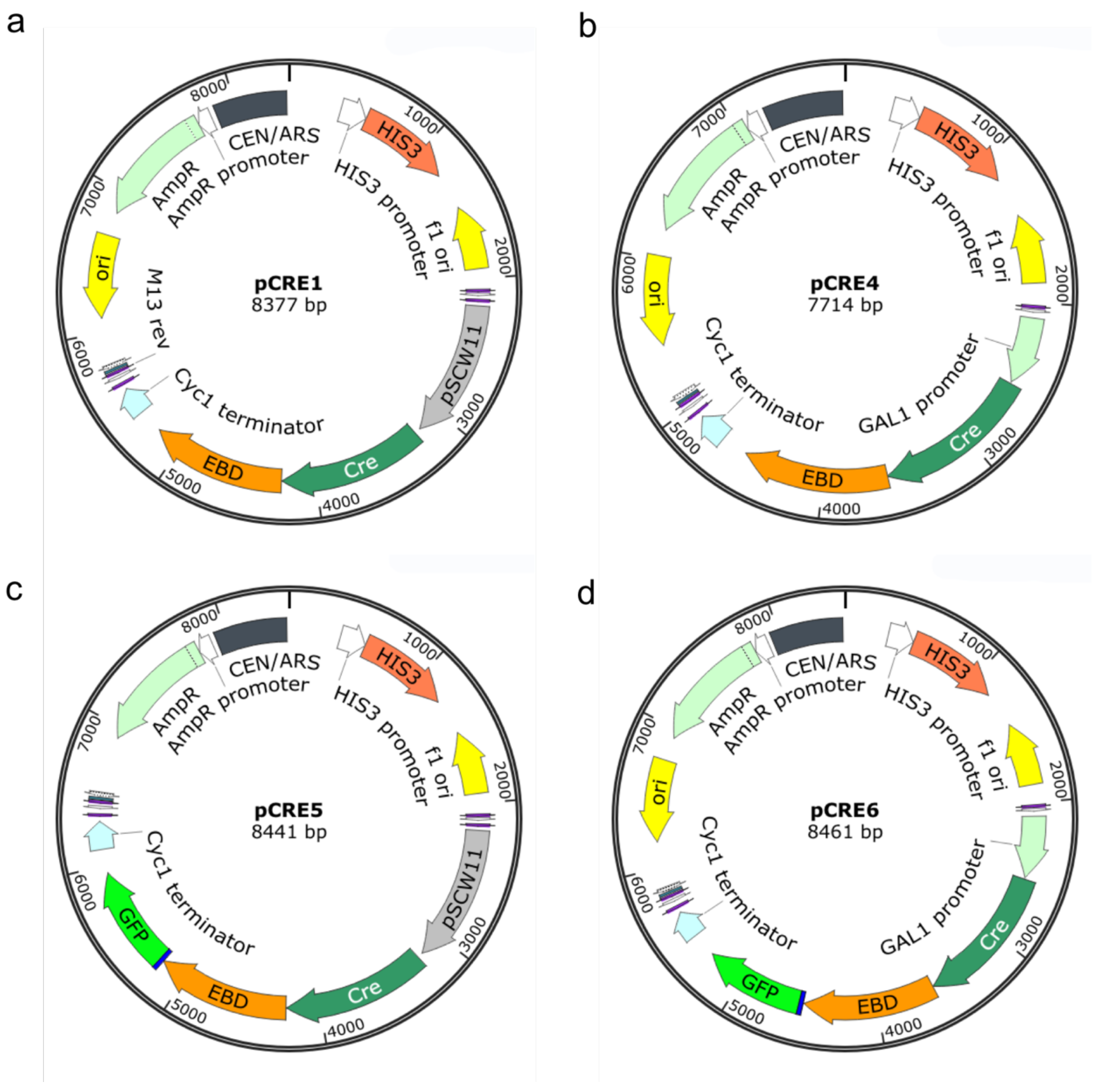

Supplementary Figure 4. Plasmids map of four CRE switches used in this study. (a) Plasmid map of the pCRE1: pSCW11-Cre-EBD-CYC1t; (b) Plasmid map of the pCRE4: pGAL1-Cre-EBD-CYC1t; (c) Plasmid map of the pCRE5: pSCW11-Cre-EBD-GFP-CYC1t; (d) Plasmid map of the pCRE6: pSCW11-Cre-EBD-GFP-CYC1t;

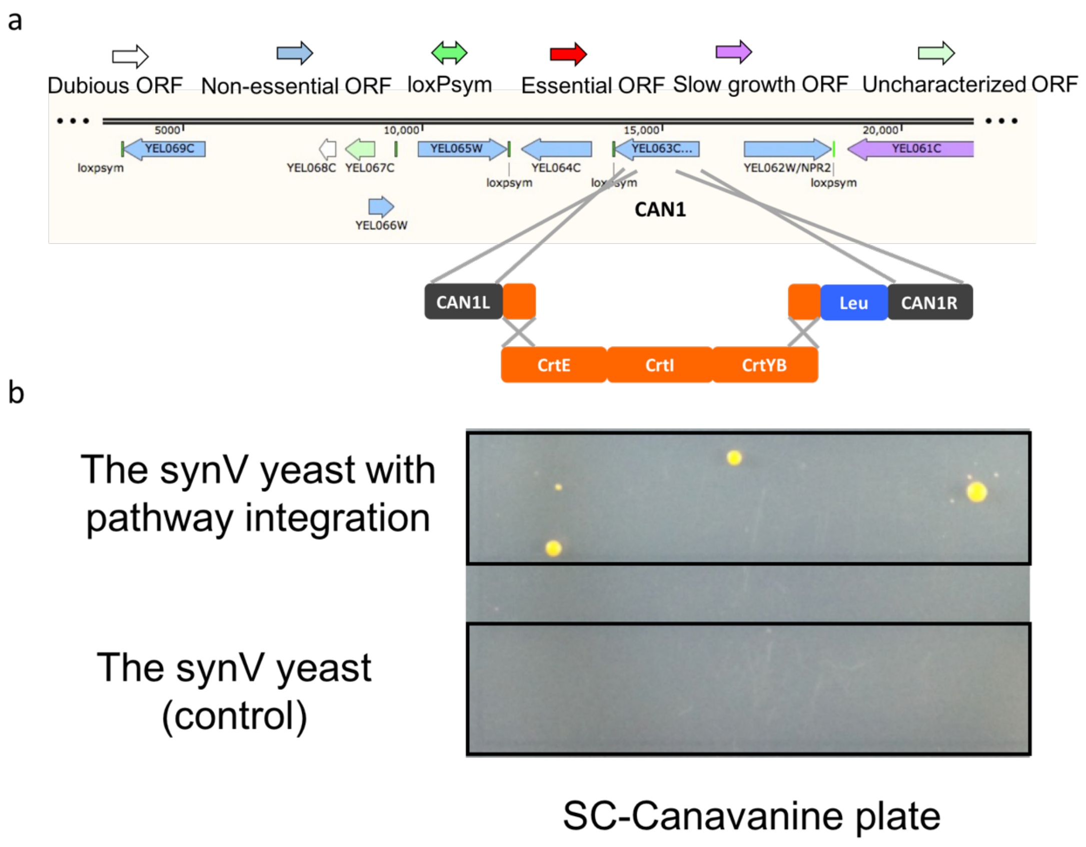

Supplementary Figure 5. Integration of the carotenoid pathway into the synV chromosome. (a) The carotenoid pathway was integrated into the YEL063C/CAN1 site with auxotrophic marker Leu2. The YEL066W, YEL065W, YEL064C, YEL063C and the YEL062W are all non-essential genes. (b) The SC-Canavanine plate was used for selection of the carotenoids pathway integration. The orange color colonies were the synV haploid yeast with carotenoid pathway integration (yJBH000) in the SC-Canavanine plate, while no colonies appear in the control group. The endogenous negative selectable gene CAN1, encoding plasma membrane arginine permease. The disruption mutant of CAN1 can grow in the presence of toxic arginine analogue L-Canavanine.

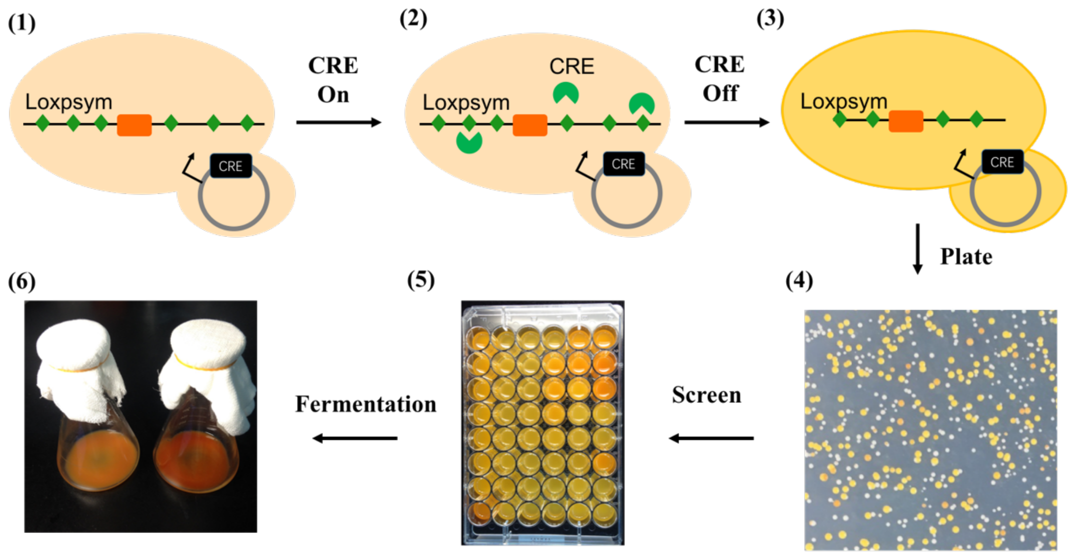

Supplementary Figure 6. Schematic diagram of the SCRaMbLE and screen system. (1) the carotenoid pathway was integrated into the chromosome synV. The parent synV strains were transformed using the tight switch pCRE4 and plated onto SC-His agar. The plates were incubated at 30°C for 72 h. (2) Single colonies were inoculated into 5 mL SC-His overnight. The cultures were washed twice with ddH_2O and re-inoculated to obtain an OD600 of 1.0 in 2% galactose SGal-His medium containing 1μM estradiol (Sigma-Aldrich). The cultures were incubated at 30°C for 8 h to turn on Cre activity in the cells and begin the SCRaMbLE progress. (3) The yeast pellet from 1 mL of culture was washed twice with ddH_2O and resuspended in 1 mL of SC-His glucose medium. (4) The SCRaMbLE library was plated on SC-His glucose agar and the strains incubated at 30°C until clear differences in carotenoid pigmentation were observed (72 h to 120 h). (5) Colonies colored darker than the wild-type parent strains were inoculated into a 48-well plate on SC-His glucose medium and dropped on SC-His glucose agar plates at 30°C for 48 h. (6) Strains colored darker than the wild-type parent strain in the 48-well plate were prepared for fermentation. Three independent colonies of each were inoculated into 5 mL of YPD medium for 24 h, then re-inoculated to obtain an OD600 of 0.1 in 40 mL YPD with 40 g/L glucose medium in 250 mL flasks, which were then incubated for 60 h at 30°C.

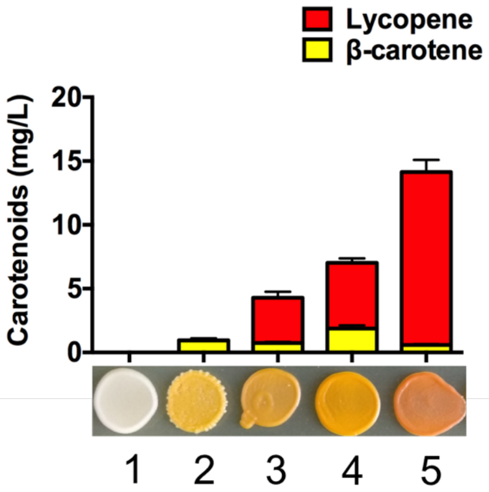

Supplementary Figure 7. Color-base screen method for high throughput screening from the SCRaMbLE library. Five scrambled colonies exhibited different colors were resuspended in 10 μL of water and dropped on an SC-His glucose plate at 30°C for 48 h. These five colonies were also inoculated in 40 mL YPD with 40 g/L glucose medium in 250 mL flasks for 60 h. Error bars represent s.d. from three independent experiments.

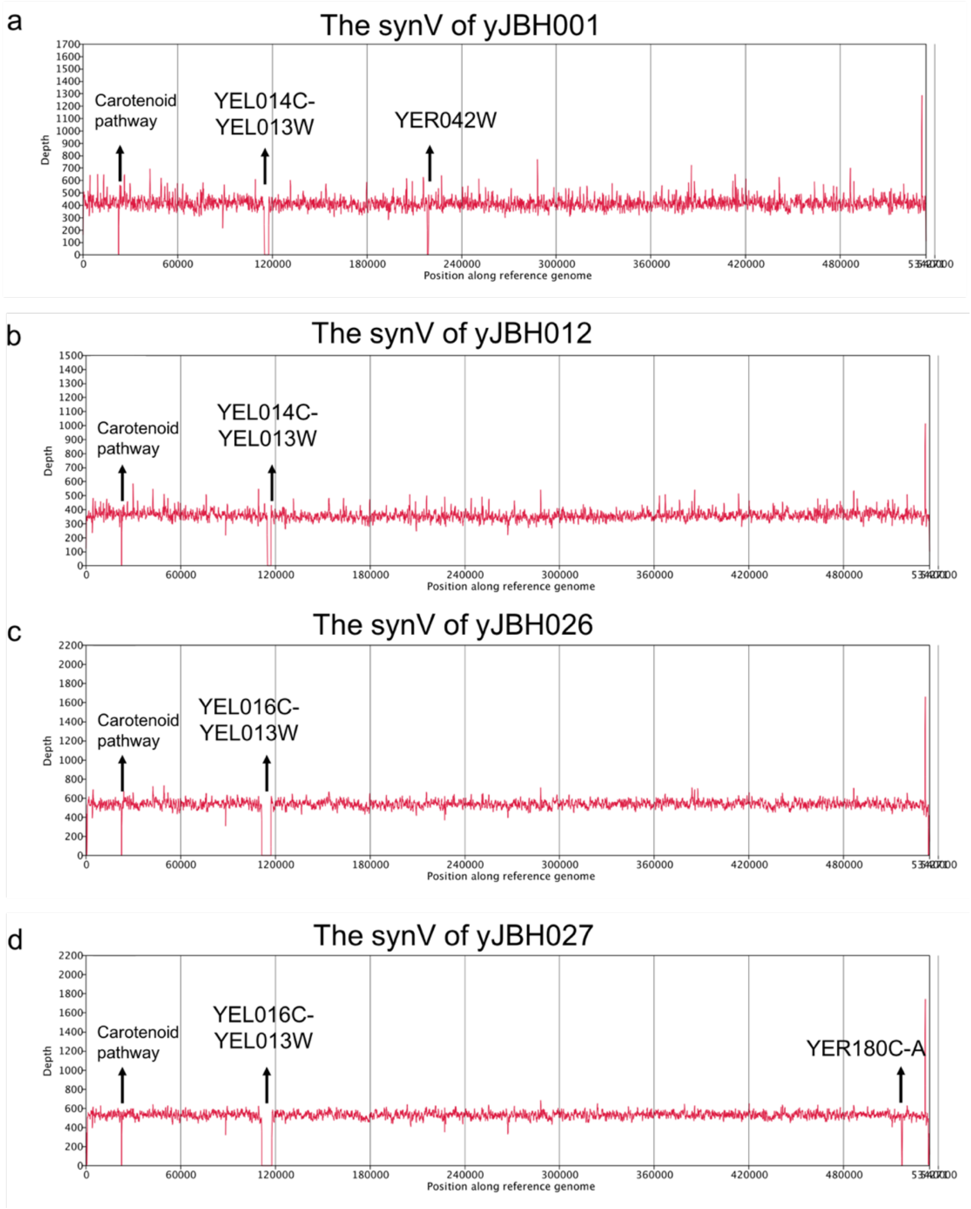

Supplementary Figure 8. Deep sequencing analysis of four haploids with high production of carotenoids. **(a)** Deep sequencing coverage of yJBH001 strain revealed two deletions of synthetic fragments (the YEL014C-YEL013W and the YER042W). (b) Deep sequencing coverage of yJBH012 strain revealed a deletions of synthetic fragments (YEL014C-YEL013W). (c) Deep sequencing coverage of yJBH026 strain revealed a deletions of synthetic fragments (YEL016C-YEL013W). (d) Deep sequencing coverage of yJBH027 strain revealed a deletions of synthetic fragments (YEL016C-YEL013W and YER180C-A). The first deletion is the YEL063C disrupted by integration of the carotenoid pathway.

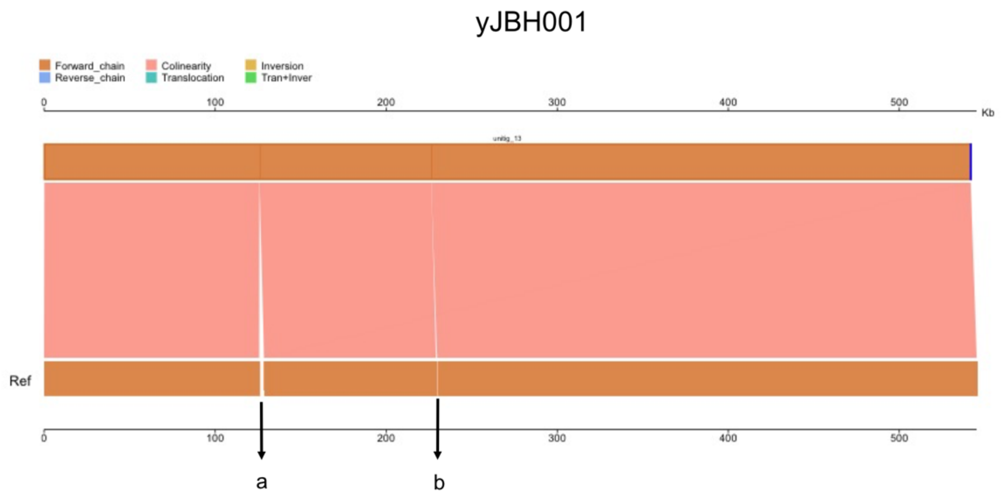

Numbers	Structure Variety	Copy Variety	Range	Size bp
a	Deletion	-1	YEL014C-YEL013W	2345
b	Deletion	-1	YER042W	819

Supplementary Figure 9. Long-Read sequencing analysis of the synV of the yJBH001. Long-Read sequencing the synV rearrangement of the haploid yJBH001 revealed two deletions of synthetic fragments: the YEL014C-YEL013W and the YER042W. The Ref represents the chromosome sequence of synV with carotenoid pathway integration. The uniting 73 represents the assembled sequence data of the yJBH001. The “a” represents the deletion of YEL014C-YEL013W, the “b” represents the deletion of YEL042W.

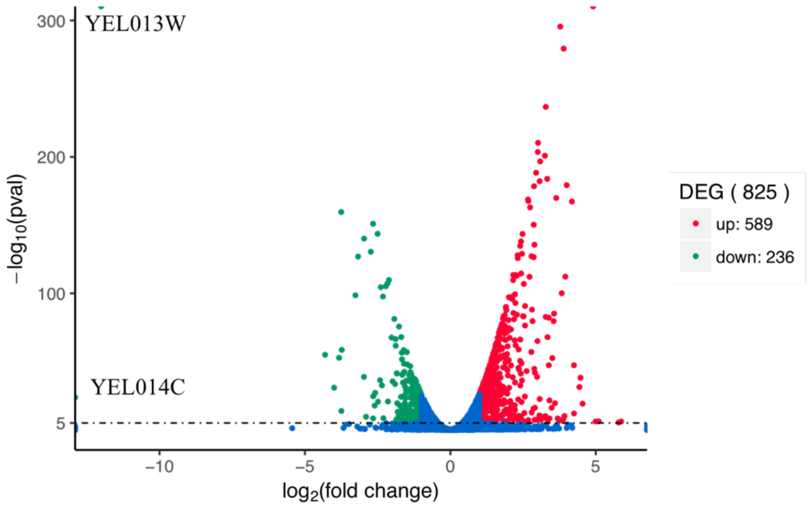

Supplementary Figure 10. Transcript profiling of ancestor (yJBH000) and scrambled yeast (yJBH012) using a volcano plot. All the strains were grown in YPD media supplemented with 40 g/L glucose at 30 °C until late-exponential phase. Three biological replicates were performed per strain. The dashed line identifies the Family Wise Error Rate (FWER) threshold at 5% (threshold = 7.02E-6). The genes significantly (Log2FoldChange > 1-fold) differentially expressed were analyzed further to identify potential mechanisms for increased carotenoids production. The YEL013W and YEL014C were deleted in the post-SCRaMbLE haploid yJBH012. Triplicate samples were used for transcriptional analysis.

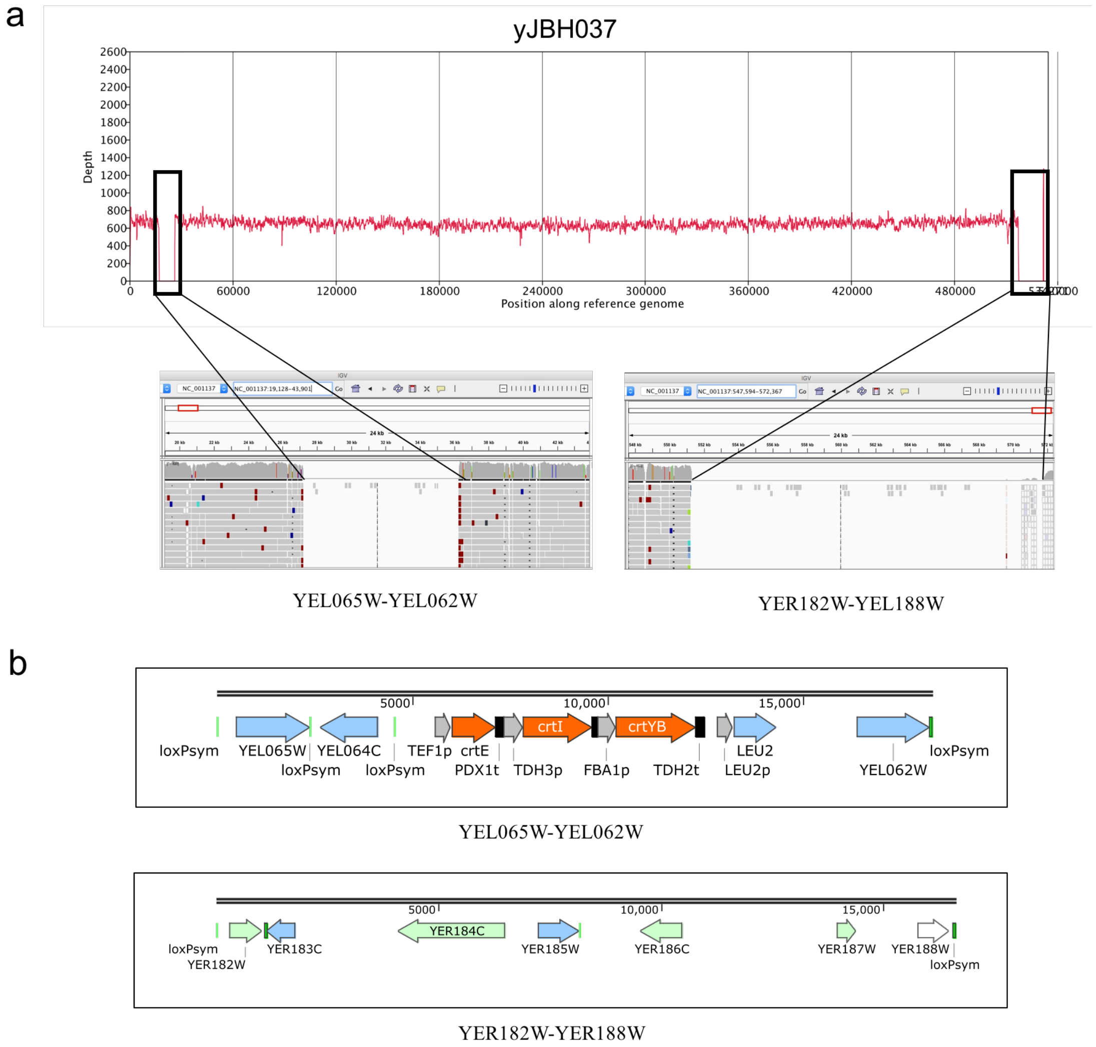

Supplementary Figure 11. Large deletion of nonessential genes. (**a**) Sequence analysis of yJBH010. Sequence data were aligned to synV. (**b**) Two larger deletion of synV were observed in yJBH010. Deletion from YEL065W to YEL062W was a 15908 bp fragments containing the carotene pathway. Deletion from YER182W to YEL188W was a 16612 bp fragments at the end of right arm of synV.

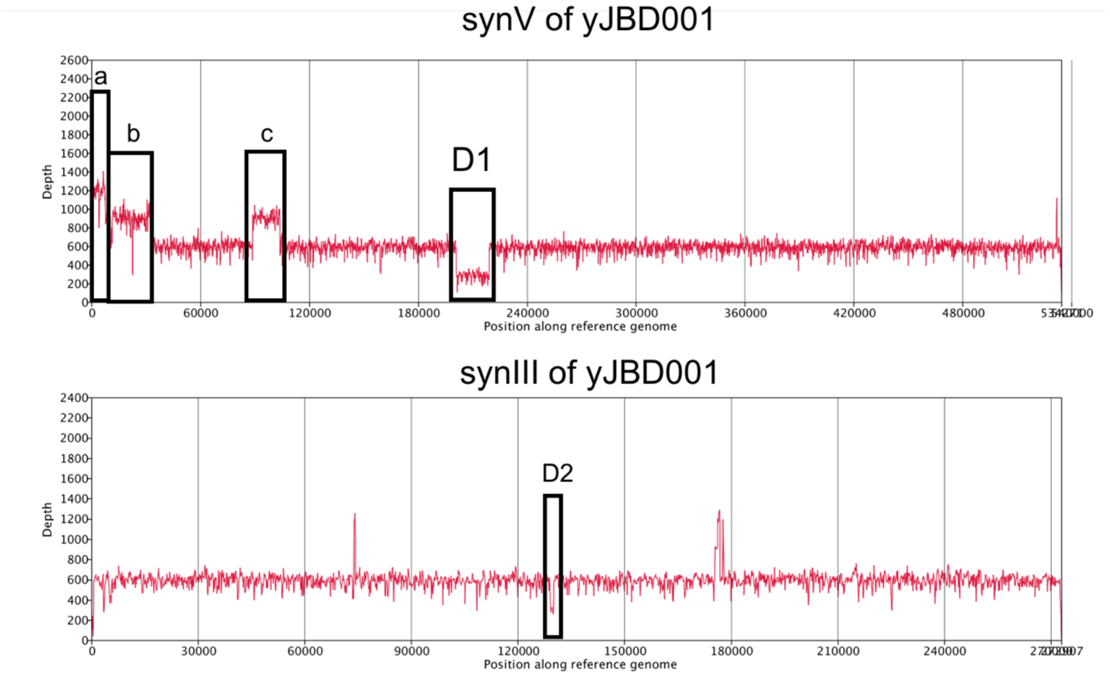

Numbers	Structure Variety	Copy Variety	Range	Size (bp)
a	Duplication	+2	YEL072W-YEL071W	6970
b	Duplication	+1	YEL070W-YEL060C	24820
c	Duplication	+1	YEL027W-YEL022W	16700
D1	Deletion	-1	YER033C-YER042W	17611
D2	Deletion	-1	YCR018C	1315

Supplementary Figure 12. Deep sequencing analysis of the synV of the yJBD001. Deep sequencing coverage of yJBD001 strain revealed three duplications (YEL072W-YEL071W, YEL070W-YEL060C and YEL027W-YEL022W) and two deletions (YER033C-YER042W and YCR018C).

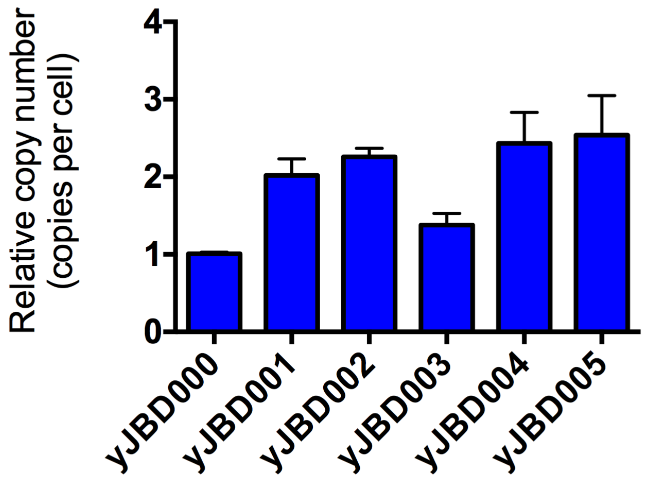

Supplementary Figure 13. Relative copy numbers assay of the carotenoids pathway in diploids. The yJBD000 were used as control. Copy numbers of carotenoids pathway of yJBD001, yJBD002, yJBD003, yJBD004 and yJBD005 were verified by qPCR. The *ALG9* gene and the *CrtE* gene were chosen as the reference gene and target genes, respectively. Error bars represent s.d. from three independent experiments.

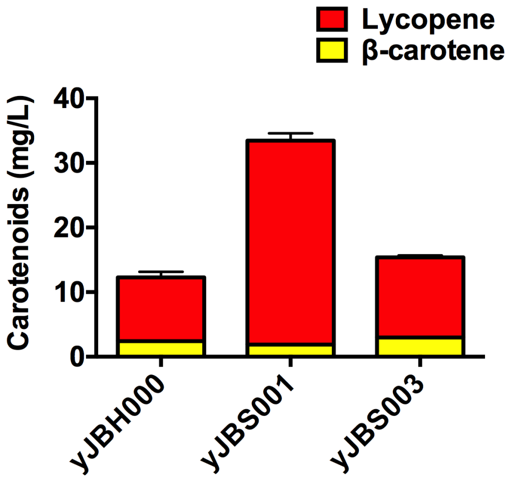

Supplementary Figure 14. HPLC analysis of extracted carotenoids from cultures of the two spores. The yJBS001 is a darker color spore and yJBS003 is a normal color spore compared with the yJBH000. Both of yJBS001 and yJBS003 were spores of the yJBD001. the yJBH000, yJBS001 and yJBS003 were 12.38 mg/L, 33.46 mg/L and 15.38 mg/L, respectively. Error bars represent s.d. from three independent experiments.

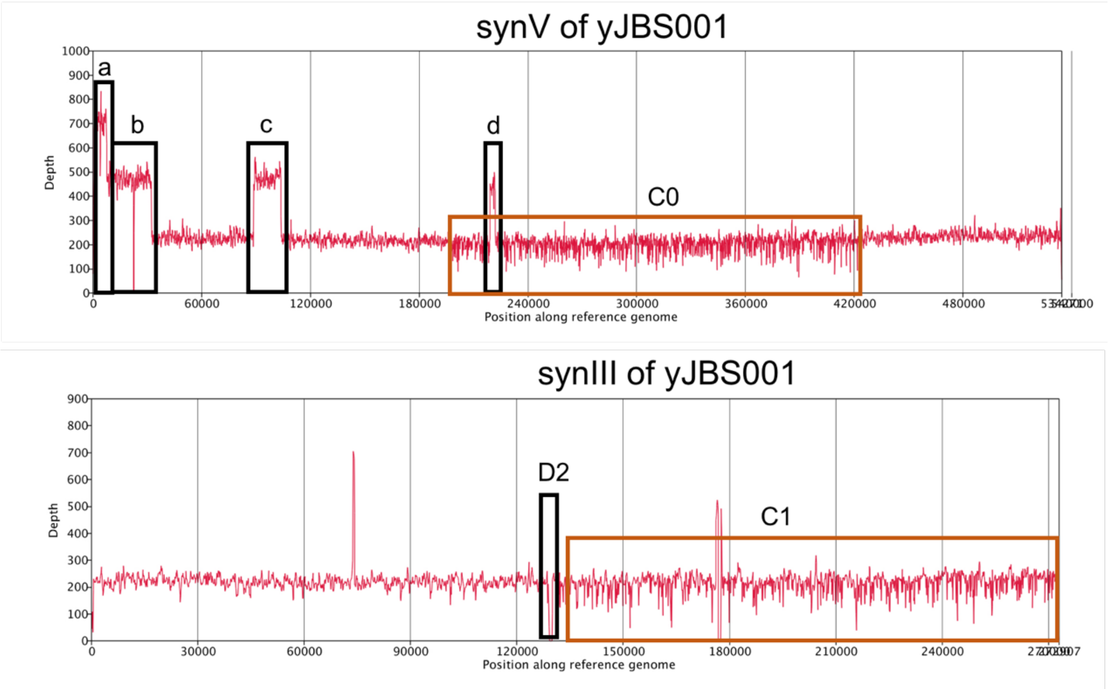

Numbers	Structure Variety	Copy Variety	Range	Size (bp)
a	Duplication	+2	YEL072W-YEL071W	6970
b	Duplication	+1	YEL070W-YEL060C	24820
c	Duplication	+1	YEL027W-YEL022W	16700
d	Duplication	+1	YER043C-YER044C	3064
C0	Crossing-over	0	YER032W-YER139C	225048
C1	Crossing-over	0	YCR018C-YCR098C	168382
D2	Deletion	-1	YCR018C	1315

Supplementary Figure 15. Deep sequencing analysis of the synV and synIII of the yJBS001. Deep sequencing coverage of synV revealed four duplications of synthetic fragments (YEL072W-YEL071W, YEL070W-YEL060C, YEL027W-YEL022W and the YER043C-YER044W) and a larger crossing-over fragment between synV and wildtype V chromosome (YER032W-YER139C). Deep sequencing coverage of synIII strain revealed a larger crossing-over fragment between synIII and wildtype III chromosome (YCR018C-YCR098C) and. Reads mapping of the right arm of the yJBS001 to wild-type chromosome III was rough while reads mapping of the right arm of the yJBS001 to synIII was smooth, which was caused by the PCR tag in the synIII.

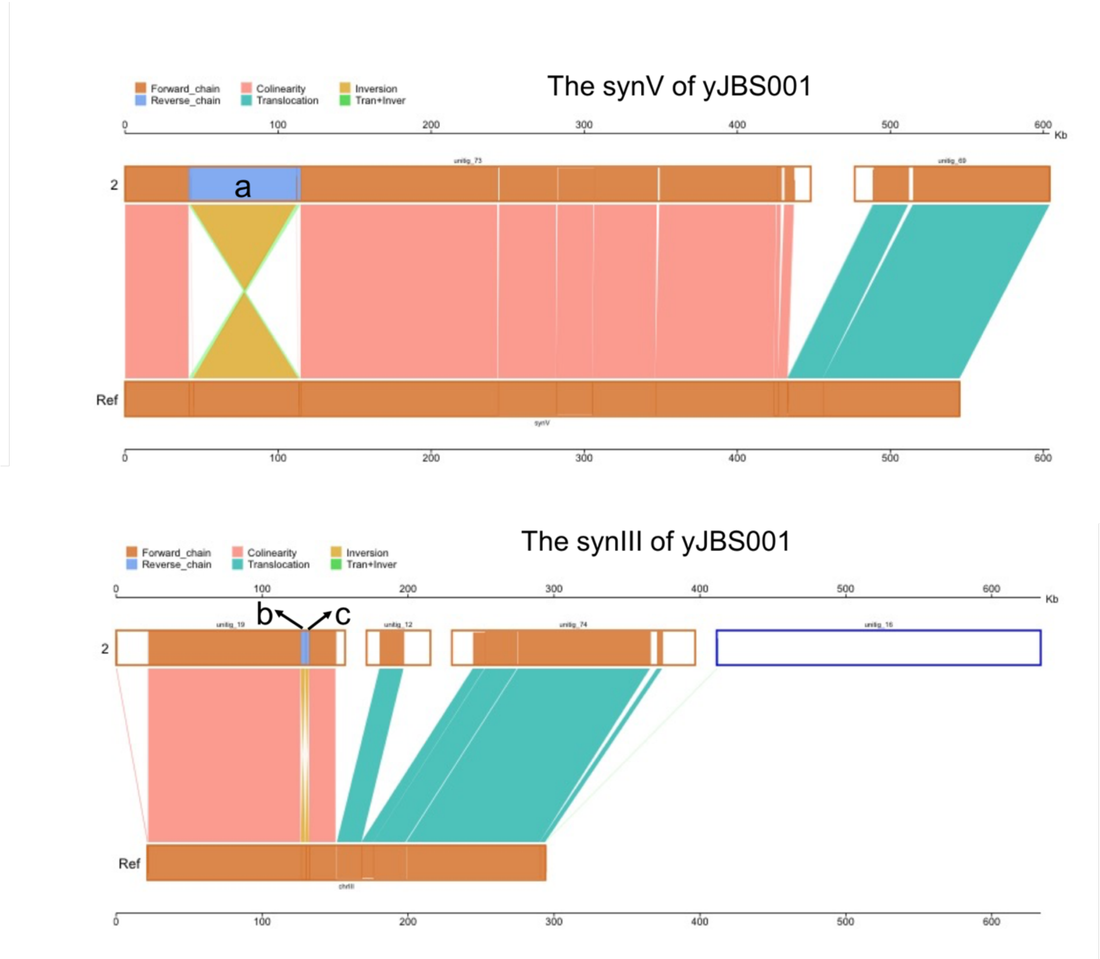

Numbers	Structure Variety	Copy Variety	Range	Size bp
a	Inversion	0	YEL059W-YEL022W	73201
b	Inversion	0	YCR007C	3284
c	Inversion	0	YCR008W	2529

Supplementary Figure 16. Long-Read sequencing analysis of the synV and the synIII of the yJBH001. Long-Read sequencing of the yJBS001 revealed one inversion on the synV (YEL059W-YEL022W) and two inversions on the synIII (YCR007C and YCR008W)

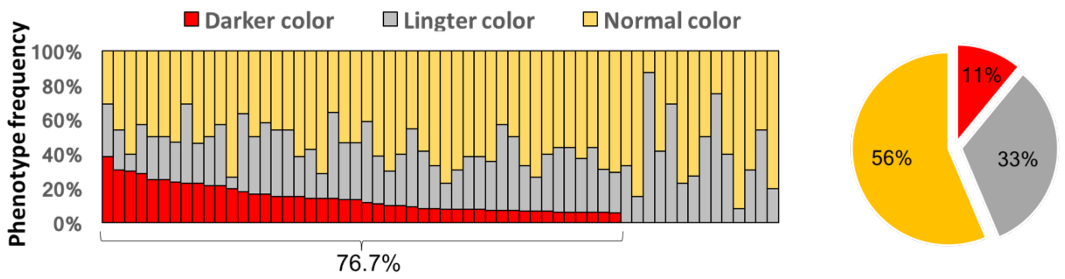

Supplementary Figure 17. Spores phenotype frequency were analyzed from counting 60 sporulation plates. Red represents darker color spores. Grey represents white color or lighter spores. Orange represents normal orange color spores. Darker color spores could be screened from 46 plates (76.7%) of the total 60 plates, and the darker color phenotype ranged from 0% to 38.5% in single plate. In sum 86 red spores (11%), 256 white spores (33%) and 442 orange spores (56%) were observed in the total 784 spores on the 60 plates, which is in accord with the law of linkage and crossing-over.

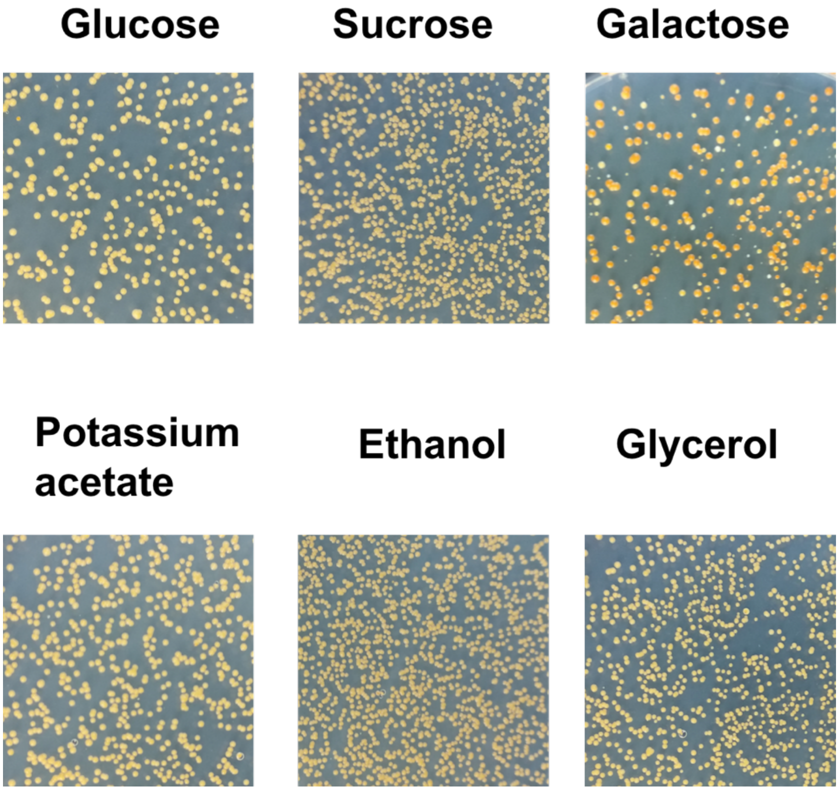

Supplementary Figure 18. Assay the leakiness of the GAL promotor in medias with different carbon source. The yJBD000 strains containing the pCRE4 were cultured for 24 hours in S-His medium containing 2% of glucose, sucrose, galactose, potassium acetate, ethanol and glycerol, respectively. white colonies appeared on the galactose medium plate.

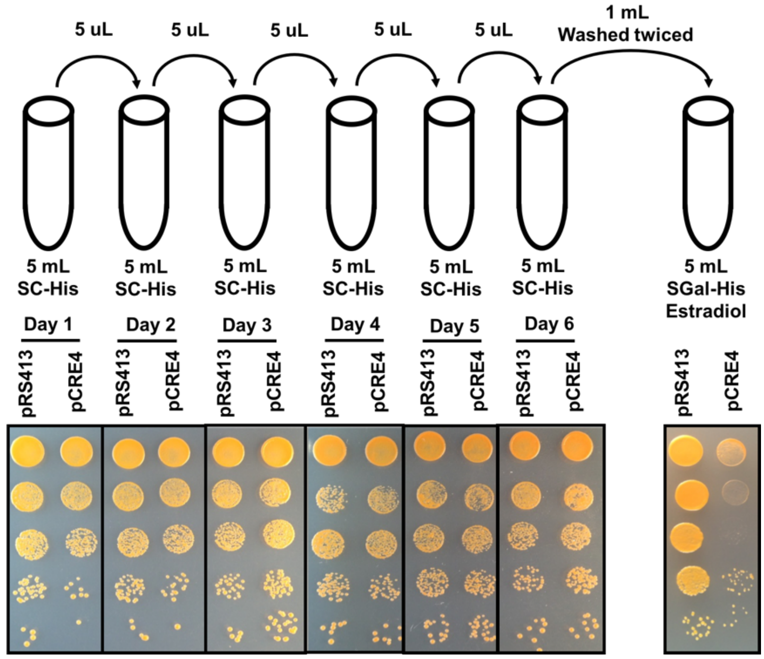

Supplementary Figure 19. Stability analysis of pCRE4 was carried out in synV haploid strains. One fresh single colony was picked up from SC-His and inoculated in 5 mL of SC-His medium at 30℃ for 24 hours, and then 5 µL of overnight culture was transferred to 5 mL of fresh SC-His medium. This experiment was continued for 5 days to get ~48 generations. Then 1 mL cells were washed twice by ddH2O and re-inoculated to an OD600 of 1.0 in 2% galactose SGal-His medium contained 1µM Estradiol (Sigma-Aldrich). Strains were incubated at 30˚C for 8 h to turn CRE activity on in cells and implement the SCRaMbLE. Ten-fold serial dilutions were carried out in water, and the dilutions corresponding to 10^{-1} to 10^{-5} spotted on the appropriate agar plates.

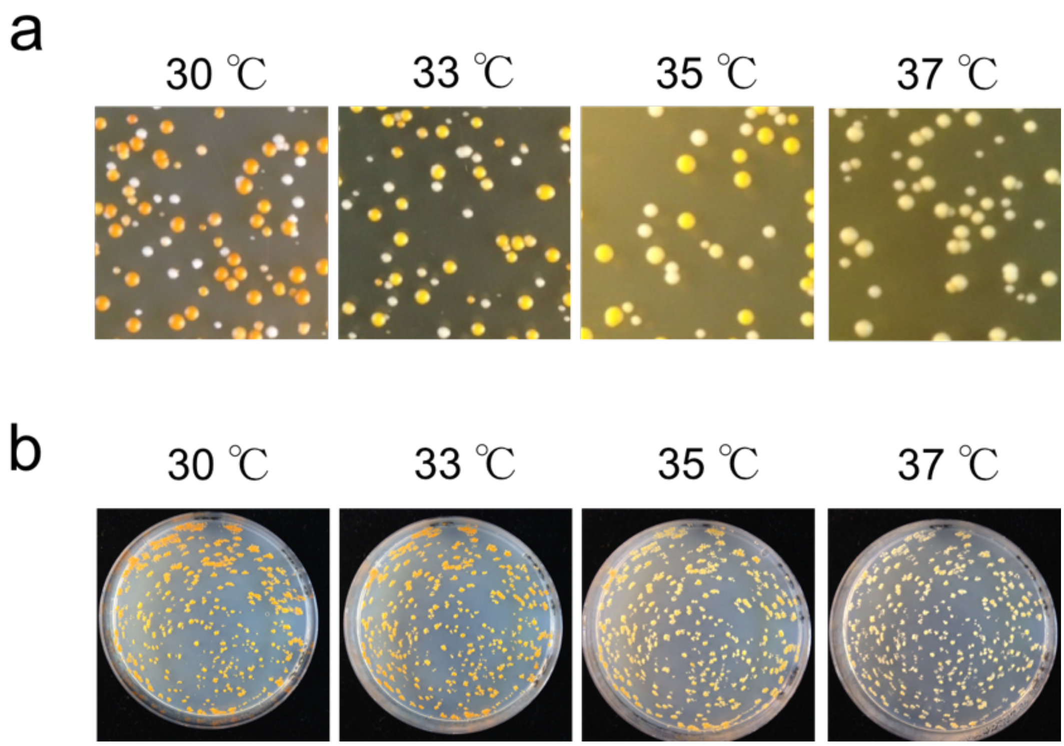

Supplementary Figure 20. High temperature assist screening. The yJBD001 were induced to SCRaMbLE. (a) The SCRaMbLEd yeast were plated on SC-His glucose agar and incubated at 30℃, 33℃, 35℃ and 37℃ for 3 days, respectively. (b) yJBD001 colonies were replicated to four plates and incubated at 30℃, 33℃, 35℃ and 37℃ for 3 days, respectively.

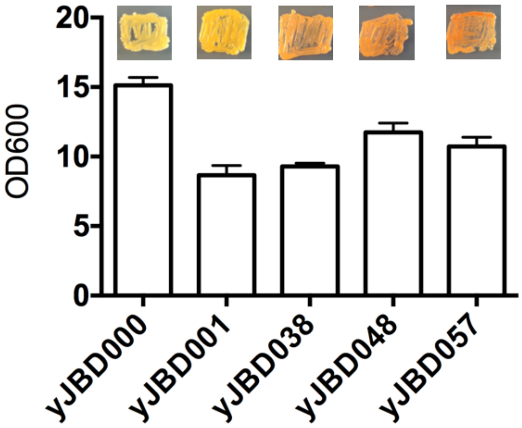

Supplementary Figure 21. Final culture ODs obtained in shake flask fermentations in YPD medium. Three independent colonies of each were inoculated into 5 mL YPD medium for 24 h, then re-inoculated to an OD600 of 0.1 in 40 mL YPD with 40 g/L glucose medium in 250 mL flasks which were then incubated for 60 h at 30℃. Error bars represent s.d. from three independent experiments.

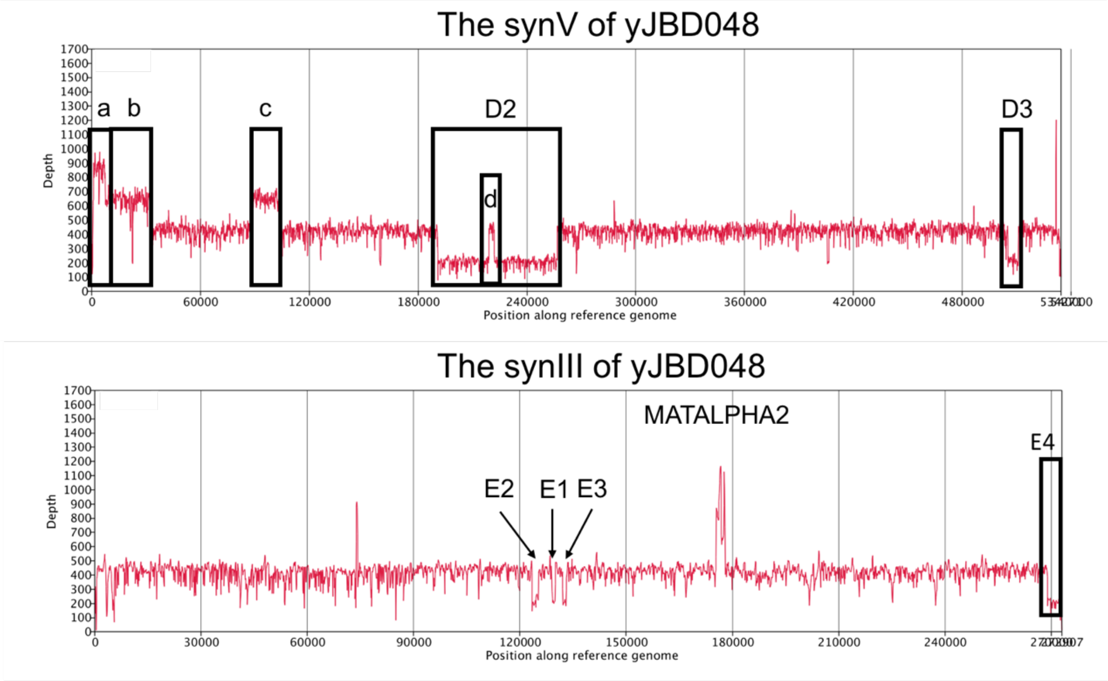

Numbers	Structure Variety	Copy Variety	Range	Size (bp)
a	Duplication	+2	YEL072W-YEL071W	6970
b	Duplication	+1	YEL070W-YEL060C	24820
c	Duplication	+1	YEL027W-YEL022W	16700
d	Translocation	0	YER043C-YER044C	3064
D2	Deletion	-1	YER026C-YER059W	65796
D3	Deletion	-1	YER175C-YER176W	5667
E1	Deletion	-1	YCR018C	1315
E2	Deletion	-1	YCR016W	1757
E3	Deletion	-1	YCR020C	886
E4	Deletion	-1	YCR098C	3682

Supplementary Figure 22. Deep sequencing analysis of the synV and synIII of the yJBD048. Deep sequencing coverage of synV revealed multiple SCRaMbLE events, including duplications of synthetic fragments (YEL072W-YEL071W, YEL070W-YEL060C, and YEL027W-YEL022W) and deletions of synthetic fragment (YER026C-TER059W and YER175C-YER176W) and a translocation (YER043-YER044C). Deep sequencing coverage of synIII revealed four deletions of synthetic fragments (YCR016W, YCR018C, YCR020C, and YCR098C).

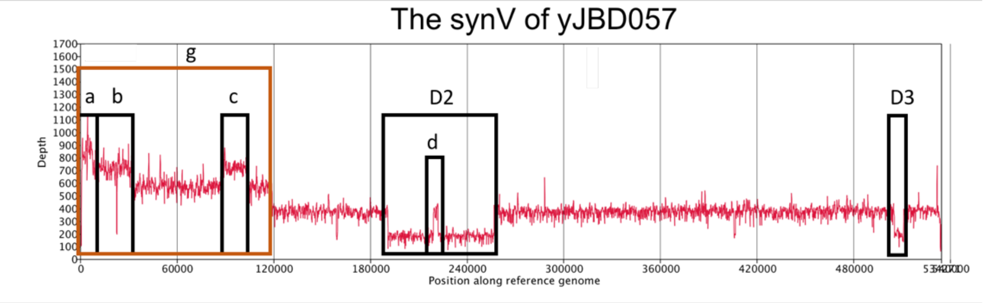

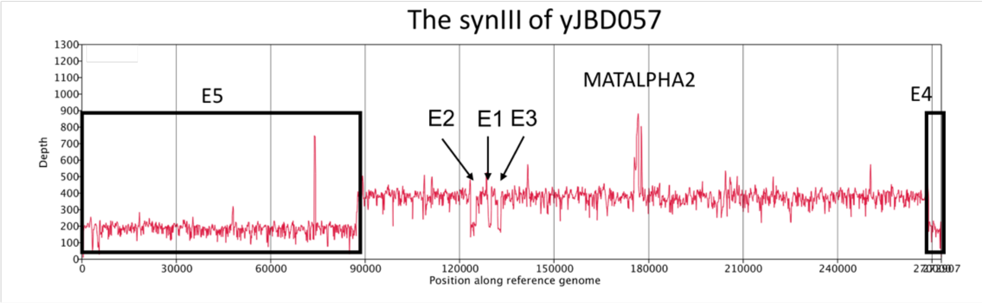

Numbers	Structure Variety	Copy Variety	Range	Size (bp)
a	Duplication	+3	YEL072W-YEL071W	6970
b	Duplication	+2	YEL070W-YEL060C	24820
c	Duplication	+2	YEL027W-YEL022W	16700
d	Translocation	0	YER043C-YER044C	3064
D2	Deletion	-1	YER026C-YER059W	65796
D3	Deletion	-1	YER175C-YER176W	5667
g	Duplication	+1	YEL072W-YEL012W	129940
E1	Deletion	-1	YCR018C	1315
E2	Deletion	-1	YCR016W	1757
E3	Deletion	-1	YCR020C	886
E4	Deletion	-1	YCR098C	3682
E5	Deletion	-1	YCL073C-YCL009C	87099

Supplementary Figure 23. Deep sequencing analysis of the synV of the yJBD057.

Deep sequencing coverage of synV revealed multiple SCRaMbLE events, including a larger duplications of synthetic fragments (YEL072W-YEL071W, YEL070W-YEL060C, YEL059C-A to TEL028W, YEL027W-YEL022W and YEL020C-B to YEL012W) and deletions of synthetic fragment (YER026C-TER059W and YER175C-YER176W) and a translocation (YER043-YER044C). Deep sequencing coverage of synIII strain revealed five deletions of synthetic fragments (YCL073C-YCL009C, YCR016W, YCR018C, YCR020C, and YCR098C).

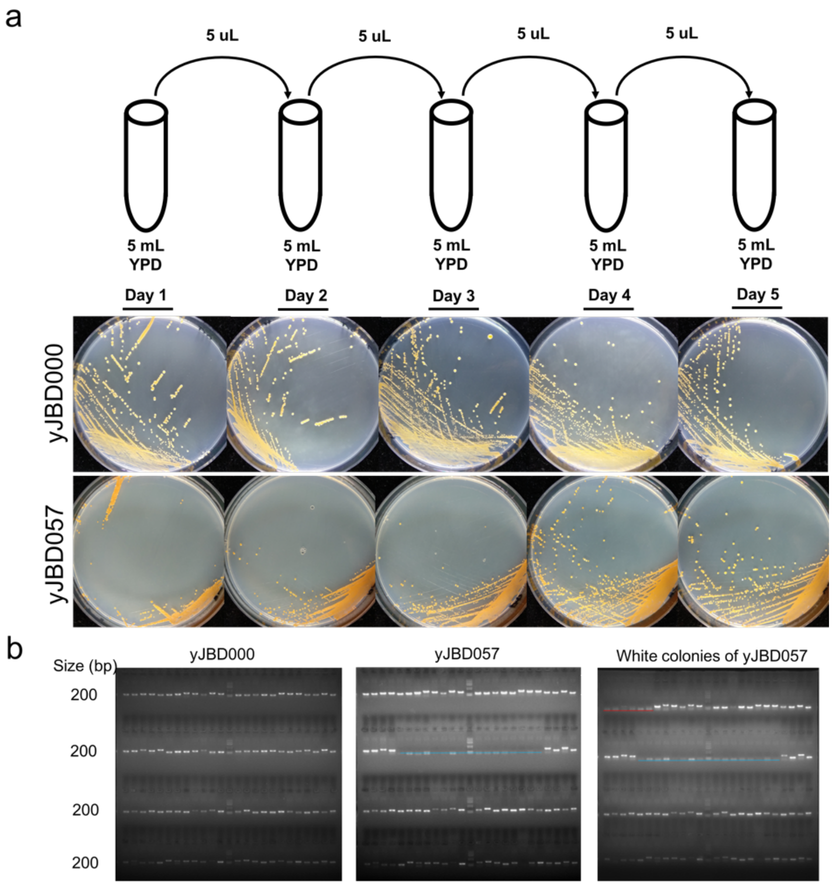

c

Strains	Number of generation	Number of total colonies	Frequency of white colonies	Deletion observed in PCRtag analysis
yJBD000	60	1784	0	None
yJBD057	60	1904	0.0068 ± 0.0031	YEL072W-YEL060C

Supplementary Figure 24. Stability assay of scrambled strains. (a) The yJBD057 and yJBD000 were serially subcultured for 5 days and streaked on YPD agar each day. Colonies from Day 5 plates were picked for PCRTag analysis. (b) PCRTag analysis of strains after 60 generations of subculture. We assayed for the loss of 96 different segments (**Supplementary Table 8**). No additional deletions were observed in yJBD000 and yJBD057, respectively. Deletions of YEL072W-YEL060C (red line) were observed in white colonies of yJBD057. Blue line indicates the deletion of YER026C-YER059W in yJBD057 as shown in **Supplementary Figure 23**. (c) Yeast cultures after 60 generations were plated on YPD agar (1:10^5 dilution) and the number of colonies of each color were counted. 0.68% of colonies observed on the yJBD057 plates were white in color, while no white colonies were observed on the yJBD000 plates.

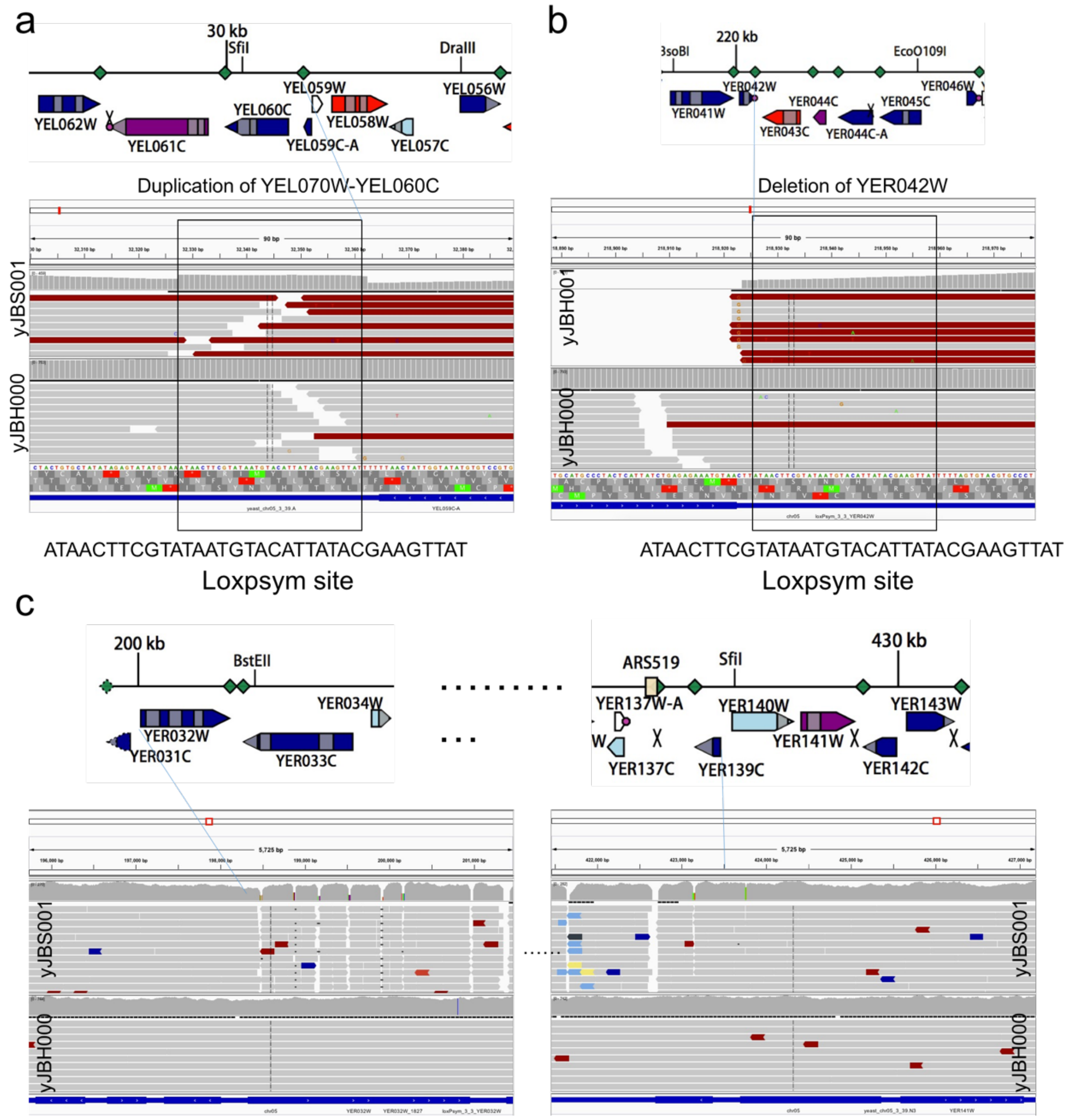

Supplementary Figure 25. Deep sequencing analysis of the boundaries of the Recombination events. (a) The right end of the YEL070W-YEL060C duplication is a loxpsym site. (b) The left end of the YER042W deletion is a loxpsym site. (c) the crossing over (YER032W-YER139C) was not flanked by loxpsym sites.

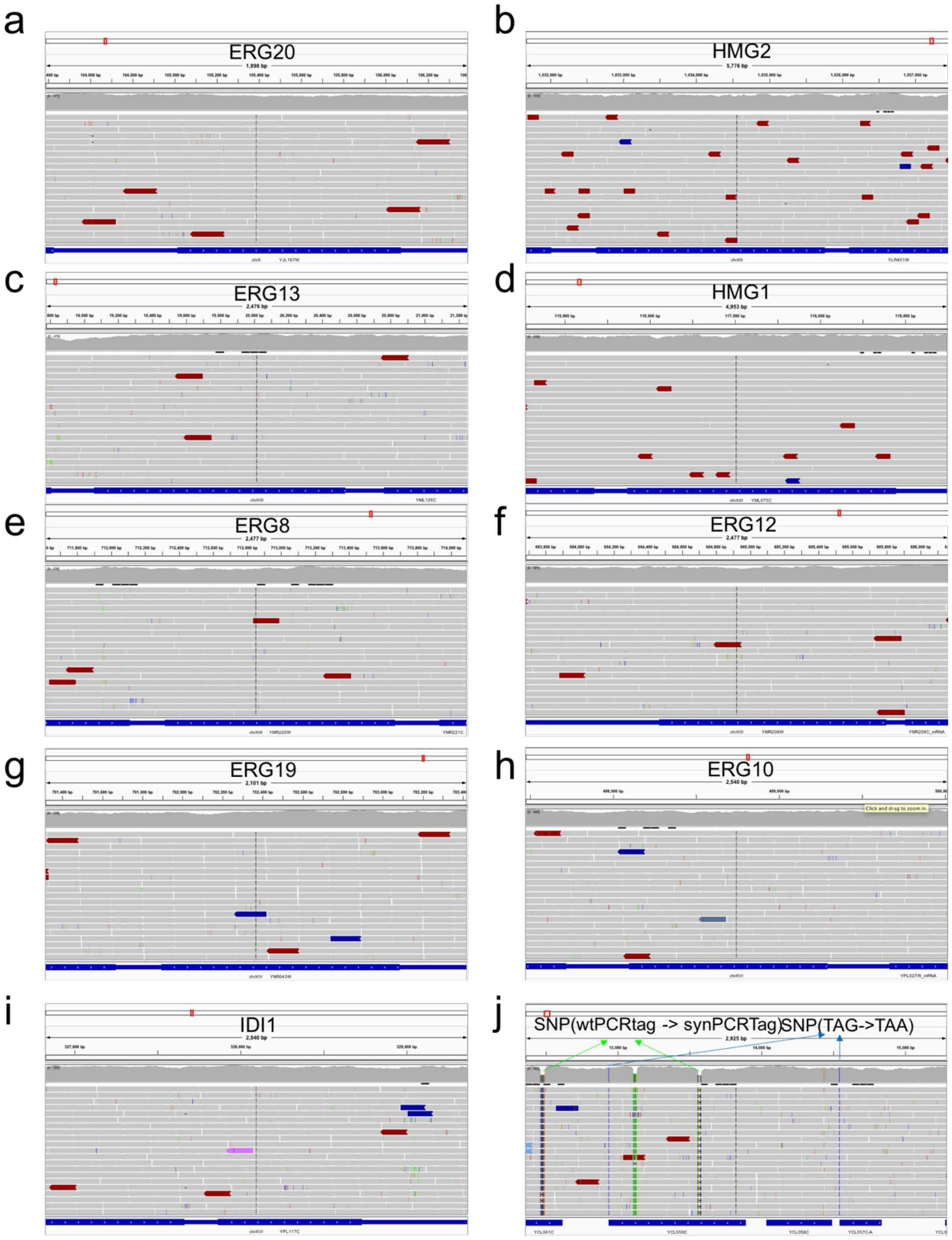

Supplementary Figure 26. Deep sequencing analysis of the non-synthetic part of the chromosome. Deep sequencing data of ERG20, HMG2, ERG13, HMG1, ERG8, ERG12, ERG19, ERG10 and IDI1 in yJBD057 were analyzed (From a to i). No no SNPs or duplications were observed in the 9 genes of the MVA pathway. (j) PCRtag and TAG-TAA switch were observed as SNP in synIII.

Supplementary Table 1. Significant transcription genes from yJBH001.

Gene	Fold Change (log2 value)	P-val	Function
PGM2	2.6201	9.14E-44	Glycolysis pathway
FBA1	1.4215	2.42E-53	
TDH1	1.6663	2.64E-38	
TDH2	1.0149	3.84E-28	
PGK1	1.5461	2.68E-56	
ENO1	2.6699	1.70E-169	
CDC19	1.3021	3.27E-45	
PDC1	1.5558	5.35E-63	
ERG10	1.3345	4.17E-43	MVA pathway
FAS1	1.1729	3.87E-35	Fatty acid biosynthesis
ACC1	1.3621	6.11E-45	

Supplementary Table 2. SCRaMbLE colonies counting in this study.

Strains used for SCRaMbLE	Lighter & white colonise	Normal colonies	Darker colonies	Total colonies	Darker colonies%
yJBH000	15	906	2	923	0.22%
yJBH000	30	765	2	797	0.25%
yJBH000	39	924	3	966	0.31%
yJBD000	491	564	8	1063	0.75%
yJBD000	821	659	7	1487	0.47%
yJBD000	798	521	8	1327	0.6%
yJBD001	220	788	6	1014	0.59%
yJBD001	208	843	8	1059	0.76%
yJBD001	188	860	7	1055	0.66%
yJBD038	20	399	3	422	0.71%
yJBD038	23	569	3	595	0.5%
yJBD038	28	782	7	817	0.86%
yJBD048	344	1032	9	1385	0.65%
yJBD048	716	608	7	1331	0.53%
yJBD048	370	692	3	1065	0.28%
yJBD057	338	673	6	1017	0.59%
yJBD057	234	400	5	639	0.78%
yJBD057	226	346	3	575	0.52%

Supplementary Table 3. Chromosome locus of genes involved in MVA pathway

Genes of MVA pathway	Chromosome
EGR20	chrX
HMG2	chrXII
ERG13, HMG1, ERG12 and ERG8	chrXIII
ERG19	chrIV
ERG10 and IDI1	chrVI

Supplementary Table 4. Yeast strains used in this study.

Numbers	*MAT*	Description	Source
**Haploid**			
synIII	α	*his3Δ1 leu2Δ0 lys2Δ0 ura3Δ0*	[1]
synV	*a*	*his3Δ1 leu2Δ0 met15Δ0 ura3Δ0*	[2]
synX	*a*	*his3Δ1 leu2Δ0 met15Δ0 ura3Δ0*	[3]
synX	α	*his3Δ1 leu2Δ0 met15Δ0 ura3Δ0*	This study
yJBH000	*a*	Haploid, synV, CAN1:: carotenoids pathway with Leu2 Marker	This study
yJBH001	*a*	SCRaMbLEd strain from the yJBH000	This study
yJBH012	*a*	SCRaMbLEd strain from the yJBH000	This study
yJBH016	*a*	SCRaMbLEd strain from the yJBH000	This study
yJBH027	*a*	SCRaMbLEd strain from the yJBH000	This study
yJBH029	*a*	SCRaMbLEd strain from the yJBH000	This study
yJBN000	*a*	yJBH000, his3D1::His3	This study
yJBN006	*a*	yJBH000, YEL014C-YEL013W:: His3	This study
yJBN007	*a*	yJBH000, YER042W:: His3	This study
yJBN008	*a*	yJBN006, YER042W:: KanMX	This study
yJBN009	*a*	yJBH000, YEL013W:: His3	This study
yJBN010	*a*	yJBH000, YEL014C:: His3	This study
**Diploid**			
yJBD000	*a*/α	Diploid, synIII & synV, wildtype III & wildtype V	This study
yJBD001	*a*/α	SCRaMbLEd strain from the yJBD000	This study
yJBD002	*a*/α	SCRaMbLEd strain from the yJBD000	This study
yJBD003	*a*/α	SCRaMbLEd strain from the yJBD000	This study
yJBD004	*a*/α	SCRaMbLEd strain from the yJBD000	This study
yJBD005	*a*/α	SCRaMbLEd strain from the yJBD000	This study

yJBN031	*a/α*	Diploid, synIII & synV, wildtype III & wildtype V, 2 copies carotenoids pathway	This study
yJBD038	*a/α*	SCRaMbLEd strain from the yJBD001	This study
yJBD048	*a/α*	SCRaMbLEd strain from the yJBD038	This study
yJBD057	*a/α*	SCRaMbLEd strain from the yJBD048	This study
yJBD069	*a/α*	SCRaMbLEd strain from the yJBD057	This study
yJBD200	*a/α*	Diploid, mating synX with yJBS001 (synIII+synV+synX)	This study
yJBD201	*a/α*	SCRaMbLEd strain from the yJBD200	This study
yJBD202	*a/α*	SCRaMbLEd strain from the yJBD200	This study
yJBD203	*a/α*	SCRaMbLEd strain from the yJBD200	This study
yJBD204	*a/α*	SCRaMbLEd strain from the yJBD200	This study
yJBD205	*a/α*	SCRaMbLEd strain from the yJBD200	This study
**Spores**			
yJBS001	*a*	Spore were dissected from the yJBD001	This study
yJBS002	*a*	Spore were dissected from the yJBD001	This study
yJBS003	*a*	Spore were dissected from the yJBD001	This study

Supplementary Table 5. Plasmids used in this study.

Name	Description	Source
pRS413	CEN/ARS with *His3* marker	[4]
pRS416	CEN/ARS with *Ura3* marker	[4]
pCRE1	pRS413, pSCW11-Cre-EBD-tCYC1	pLM006[1]
pCRE2	pRS413, pZEO1-Cre-EBD-tCYC1	This study
pCRE3	pRS413, pGAL1-Cre-tCYC1	pSH62[5]
pCRE4	pRS413, pGal1-Cre-EBD-tCYC1	pSH62-EBD[5]
pCRE5	pRS413, pSCW11-Cre-EBD-GFP-tCYC1	This study
pCRE6	pRS413, pGAL1-Cre-EBD-GFP-tCYC1	This study
pCaro	pRS416, pTEF1-crtE-tPDX1-pTDH3-crtI-tMPE1-pFBA1-crtYB-tTDH2	This study
pCAN-A	pUC19, CAN1L(left overlap)-pTEF1	This study
pCAN-B	pUC19, tTDH2-Leu2-CAN1R(right overlap)	This study

Supplementary Table 6. Primers used in this study.

Numbers	5'-3' Primer sequence	Comments
BJ001	ATAGGGCGAATTGGGTACCGGGCCCCCCCTCGAGGTCGA CACGGATTAGAAGCCGCCGA	pCRE4 GAL1p F
BJ002	GGTGTACGGTCAGTAAATTGGACATTATAGTTTTTCTCC TTGACGTTA	pCRE4 GAL1p F
BJ003	TAACGTCAAGGAGAAAAAACTATAATGTCCAATTTACTG ACCGTACACC	pCRE4 Cre-EBD-Cyct F
BJ004	CCCTCACTAAAGGGAACAAAAGCTGGAGCTCCACCGCG GTGGCGGCCGCTCTAG	pCRE4 Cre-EBD-Cyct R
BJ005	CTCACTATAGGGCGAATTGGGTACC	Promoter-Cre-EBD F
BJ006	CATACCTCCTCCGCTTCCACCTCCTCCAGCGCTGACTGTG GCAGGGAAACCCTCT	Promoter-Cre-EBD R
BJ007	GCTGGAGGAGGTGGAAGCGGAGGAGGTATGCGTAAAGG AGAAGAACTTTTCACTG	GFP F
BJ008	ATATTGAGTCAATATCAGGCATTCTACTCATTATTTGTAT AGTTCATCCATGCCA	GFP R
BJ009	TGAGTAGAATGCCTGATATTGACTC	CYC1 terminator F
BJ010	CCCTCACTAAAGGGAACAAAAGCTG	CYC1 terminator F
Carotenoid		
BJ011	CCTCGAGGTCGACGGTATCGATAAGCTTGATATCGAATT CACAATGCATACTTTGTACG	TEF1p F
BJ012	ACTCGAGTGGAATTGCTGTGAGGATGTTCGCGTAATCCA TTTTGTAATTAAAACTTAGA	TEF1p R
BJ013	ATGGATTACGCGAACATCCTC	CrtE F
BJ014	TCACAGAGGGATATCGGCAAG	CrtE R
BJ015	GGAAGCGATCCTGAAAAAGCTTGCCGATATCCCTCTGTG AATAAAAAACACGCTTTTTC	TDH3p F
BJ016	TGATAGCTGTGGGTTTATCCTGATCTTGTTCTTTTCCCAT TTTGTTTGTTTATGTGTGT	TDH3p R
BJ017	ATGGGAAAAGAACAAGATC	CrtI F
BJ018	TTATTCAGAAAGCAAGAACAC	CrtI R
BJ019	GTGATCGCTCGATCCGTTGGTGTTCTTGCTTTCTGAATAA CAATACTGACAGTACTAAA	FBA1p F
BJ020	TATAGATCAGATGGATCTGGTAATATGCGAGAGCCGTCA TTTTGAATATGTATTACTTG	FBA1p R
BJ021	ATGACGGCTCTCGCATATTAC	CrtYB F
BJ022	TTACTGCCCTTCCCATCCGC	CrtYB R
BJ023	AGTCTTGAGTGTGGTCATGAGCGGATGGGAAGGGCAGT AAATTTAACTCCTTAAGTTAC	TDH2t F

BJ024	CCGCTCTAGAACTAGTGGATCCCCCGGGCTGCAGGAATT CTGATCACGGCTAAAACGGT	TDH2t R
BJ025	CAGCTATGACCATGATTACGCCAAGCTTGCATAAATCTG ATGTGCGAGATTGAG	CAN1-A F
BJ026	GAACGTACAAAGTATGCATTGTGAATTCAGCTGCAAACC CCAGAAAATCCGTTC	CAN1-A R
BJ027	GAACGGATTTTCTGGGGTTTGCAGCTGAATTCACAATGC ATACTTTGTACGTTC	TEF1 F
BJ028	GTTGTAAAACGACGGCCAGTGAATTCGAGCTCTTTGTAA TTAAAACTTAGATTAG	TEF1 R
BJ029	AAACAGCTATGACCATGATTACGCCAAGCTTATTTAACT CCTTAAGTTACTTTAATG	TDH2t F
BJ030	TGATCACGGCTAAAACGGTCGAATG	TDH2t R
BJ031	CATTCGACCGTTTTAGCCGTGATCAGAATTCTGCATGCCT GCAG	Leu2 F
BJ032	CCAAGTCATTCAATTTTGGACGTACGCTCGGTACCCGGG GATCCAATACG	Leu2 R
BJ033	CGTATTGGATCCCCGGGTACCGAGCGTACGTCCAAAATT GAATGACTTGG	CAN-B F
BJ034	TTGTAAAACGACGGCCAGTGAATTCGAGCTCCACAAACA CACCACAGACGTGGGTC	CAN-B R
BJ035-1	ACTGTGATCTTTTCGTCACTGACGGGTCCCTGCTATTAGA TTTGTAAACCTGTGCGGTATTTCACACCGC	Delete YEL014C-His F
BJ035-2	CAGAAAGCAAAGCAGACTCACACAAAAATTGATCACA ATGACAGCACTAGCAGATTGTACTGAGAGTGCACC	Delete YEL014C-His R
BJ036-1	GCAAACTATAAGGGTGTTCTTTCTTCTGTACTATATATAC ATTTGCAACTTGTGCGGTATTTCACACCGC	Delete YEL013W-His R
BJ036-2	AGAATAGTGTTGATATATGATAAAATTATTGTGGAAATC AATAATTAAGAGCAGATTGTACTGAGAGTGCACC	Delete YEL013W-His R
BJ037	ACCGGTTTGTAGCCTGTGACAGTGATAGCAGTAGCACTA TTGAATGAGTTTGTGCGGTATTTCACACCGC	Delete YER042W-His F
BJ038	TCATTCATGCACTTGACTTTTTTTCATAAATAAGGGCACG TACACTAAAAGCAGATTGTACTGAGAGTGCACC	Delete YER042W-His R
BJ039	ACCGGTTTGTAGCCTGTGACAGTGATAGCAGTAGCACTA TTGAATGAGTTGACATGGAGGCCCAGAATA	Delete YER042W-G418 F
BJ040	TCATTCATGCACTTGACTTTTTTTCATAAATAAGGGCACG TACACTAAAAAGTATAGCGACCAGCATTC	Delete YER042W-G418 R
BJ041	AATGATAAGGATCAGGTACTAGC	Test YEL014C-YEL013W F
BJ042	CGTAATTGCAATGTGGCAGC	Test YEL014C-YEL013W R
BJ043	GATTCTATGCACGAGATGTTTC	Test YER042W-His F
BJ044	GCCACTGATGTCATGTTGGC	Test YER042W-His R
BJ045	GGTCTCAAAAATGAGCAGTAGGTGTCTCGGG	YER033C3 WT forward

BJ046	GGTCTCTCAGTGACGCTAGGCCAGTACCTCC	YER033C3 WT reversed
BJ047	GGTCTCAAAAAGTGGTCAGGCCTTGTCAATT	YER033C3 synthetic forward
BJ048	GGTCTCTCAGTCACAGGGCGGGAAATAAGCT	YER033C3 synthetic reversed
BJ049	GCATTCTGCAGTCGCCAATGCAGAGTTG	YER034W1 WT forward
BJ050	ATCGGTCTCTAGTTCTTCCGGTAGATCG	YER034W1 WT reversed
BJ051	TCACAGCGCTGTTGCTAACGCTGAGTTA	YER034W1 synthetic forward
BJ052	GTCAGTCTCCAATTCTTCTGGCAAGTCA	YER034W1 synthetic reversed
BJ053	GGTCTCAAAAACGGACTATGCCCGAGTGGT	YER036C1 WT forward
BJ054	GGTCTCTCAGTGCTCGGCGCGTAGCTTCA	YER036C1 WT reversed
BJ055	GGTCTCAAAAAGTATTTCTGTGAGATGATTATTTTC	YER036C1 synthetic forward
BJ056	GGTCTCTCAGTAGGGAGAAAAGGAACTTATAACGG	YER036C1 synthetic reversed
BJ057	GGTCTCAAAAAAAGTGAGAATGTATCCGGCGAG	YER038C1 WT forward
BJ058	GGTCTCTCAGTAAGTATGGCACCAAACCCTTGG	YER038C1 WT reversed
BJ059	GGTCTCAAAAAAGTAGAAGTTAAAGAAGAACAGGAGG	YER038C1 synthetic forward
BJ060	GGTCTCTCAGTAGCCCGTGAATGAAAGAGGA	YER038C1 synthetic reversed
BJ061	AAGTGCAGGAAATAGTGTCGCATCCTCA	YER040W2 WT forward
BJ062	AGGTCTTGAGACACTCGGTGAATCTACA	YER040W2 WT reversed
BJ063	TTCAGCTGGTAACTCAGTTGCTAGTAGC	YER040W2 synthetic forward
BJ064	TGGACGGCTAACTGATGGGCTGTCAACT	YER040W2 synthetic reversed
BJ065	GGTCTCAAAAATCTCTAGCCTCATCAGCTCCG	YER041W2 WT forward
BJ066	GGTCTCTCAGTAATGTAGATTGGGTGGCTGCT	YER041W2 WT reversed
BJ067	GGTCTCAAAAAGAAAAGCTGCCAACGTGACC	YER041W2 synthetic forward
BJ068	GGTCTCTCAGTGTACGACAACTTACTCTTAATCGG	YER041W2 synthetic reversed
BJ069	GGTCTCAAAAAACTACGGCGAATTCGCTGAC	YER042W1 WT forward
BJ070	GGTCTCTCAGTTCATCATCTTCACGATCTTCAGCATC	YER042W1 WT reversed
BJ071	TTCTAGGTTGGTGCATTGAG	YER042W1 synthetic forward
BJ072	GTGGGCAAATAAACCTGATCTGTACTGG	YER042W1 synthetic reversed
BJ073	GGACAGATAGCGTAGAGA	ALG9 F
BJ074	TGTGGAATTATTGCCTTCT	ALG9 R
BJ075	GAGATTATCAGCAATATCAATG	YEL071W F
BJ076	GTAACGACACCGATAGTA	YEL071W R
BJ077	ATATCGTGCTCCTTGAAC	crtE F
BJ078	TTGACATCCAACCAATAGT	crtE R
BJ079	GACTATTGAGCCTGTTAATGTG	YEL022W F
BJ080	TCTTCTTGACTTCCTTCTTCTT	YEL022W R
BJ081	TACTCTTCTTCAGTTTCA	YER043C F
BJ082	CCTCTTGTAACATCTATTC	YER043C R
BJ083	TCAATATGCTCAGGAATAGGA	YER036C F
BJ084	GCCGTAGATATGGTCTTCT	YER036C R

Supplementary Table 7. Deletions/duplications observed in this study

Strains	Structure variety	Copy variety	Total copy numbers	Range	Size (bp)
yJBH001	Deletion	-1	0	YEL014C-YEL013W	2345
	Deletion	-1	0	YER042W	819
yJBH012	Deletion	-1	0	YEL014C-YEL013W	2345
yJBH026	Deletion	-1	0	YEL016C-YEL013W	5933
yJBH027	Deletion	-1	0	YEL016C-YEL013W	5933
	Deletion	-1	0	YER180C-A	819
yJBH037	Deletion	-1	0	YEL065W-YEL062W	18304
	Deletion	-1	0	YER182W-YER188W	16612
yJBS001	Duplication	2	3	YEL072W-YEL071W	6970
	Duplication	1	2	YEL070W-YEL060C	24820
	Duplication	1	2	YEL027W-YEL022W	16700
	Duplication	1	2	YER043C-YER044C	3064
	Deletion	-1	0	YCR018C	1315
	crossing-over	0	1	YER032W-YER139C	225048
	crossing-over	0	1	YCR019W-YCR098C	168382
yJBD001	Duplication	1	2	YEL070W-YEL060C	24820
	Duplication	1	2	YEL027W-YEL022W	16700
	Deletion	-1	0	YER033C-YER042W	17611
	Deletion	-1	0	YCR018C	1315
yJBD048	Duplication	2	3	YEL072W-YEL071W	6970
	Duplication	1	2	YEL070W-YEL060C	24820
	Duplication	1	2	YEL027W-YEL022W	16700
	Translocation	0	1	YER043C-YER044C	3064
	Deletion	-1	0	YER026C-YER059W	65796
	Deletion	-1	0	YER175C-YER176W	5667
yJBD057	Duplication	3	4	YEL072W-YEL071W	6970
	Duplication	2	3	YEL070W-YEL060C	24820
	Duplication	2	3	YEL027W-YEL022W	16700
	Translocation	0	1	YER043C-YER044C	3064
	Deletion	-1	0	YER026C-YER059W	65796
	Deletion	-1	0	YER175C-YER176W	5667
	Duplication	1	2	YEL059C-A-YEL028W	56536
	Duplication	1	2	YEL020C-B- YEL012W	15325

Supplementary Table 8. Synthetic PCRTags used in this study

Number	Amplicon	Forward Syn	Reverse Syn
1	YEL071W_amp1	CAGCGGTAGTAACAAACGTCATGATGAC	ATTCAAACGCTCGGTAACGGCAGCGCTA
2	YEL070W_amp1	TAGTAACCCTGCTATCCAGGATACCGTT	ATGTGGGTCCTTGCCACCTTTGACAGCA
3	YEL067C_amp1	GACGGCGCTAACGAAGATAGTGCTACAT	TTGCAGCAGAAGATTCCCACCAGACAAT
4	YEL065W_amp2	CATCTTACCATTGGCCTGCATCCCATTG	AACAACGACCCAGCCGATAACTTCTGGA
5	YEL064C_amp1	TGAAAAGATGGCACGCAAAACATGTGGG	TGCTTTGGGTTTCATCGTCGACTGGACC
6	YEL063C_amp1	ACTCTGGGTAACGCTGTAAGCTAAGCTG	CCGTGTTAACGGTGAGGACACCTTTAGC
7	YEL061C_amp1	ACCATCGAAACCGGTGTTTGAGCTACCA	CGAAGTTGCTGGTCCTTTGTTTCAGGAC
8	YEL060C_amp2	GCTAGTGCCGCTCAAGGTAGCAGTA	TGGCGTTGCCAAGAACGCTAATGTCGTC
9	YEL052W_amp1	CGGCTTGAAGAGTGTTTTCTCAAGAGGT	AGCAACATCGGTAACTTGGAACTCATCG
10	YEL050C_amp1	TAACAAAGCAATGTGGCTACTACGGCCA	TAGCCCAGGCTTACGTTGGTATAGAAGT
11	YEL047C_amp1	CTTCCATGAGCTTGATCTATCGTTAGGG	CCAATTAGGCGGTCATAGCGTTGCTCGT
12	YEL044W_amp1	CGGTTTCCCAAGCCGTTTTAAAAGCGCT	AGCACCACGTAACTTCAAATACTCCTGG
13	YEL043W_amp2	TCACGCTAGCAGCCCTCCATTTAATAGT	GTTGGCTGGGCTGCTAGCGCTTTTATGT
14	YEL042W_amp1	CAGTGTCGGCGCTGCCAATAGTTTG	ACCCATAATACTGACGCCGTCGCCTTCA
15	YEL041W_amp1	CGGTAGTACCGCTTATAGCTTATCAGCT	AACGCTGTATGGTGAAGCGGTGATAACG
16	YEL040W_amp1	AAGCACCGCTACTAGCAGTAGTAAGACC	GCCAGCATTGTTGCCTGACATGCTTGAG
17	YEL038W_amp1	TCCAGCTCACGACAGCTTAGACTTAAAC	AACTGGAGCGTTGCCTGGTCTACTG
18	YEL037C_amp1	GGTCAAACCGATACTGCCAGGTGGA	CGCCCCAGAAGGTAGCCAACCA
19	YEL030W_amp2	TGCTCGTATCACTGCTAGCGACATCAGC	AACCAACTCTCTCTCGCCTTGAAAGACC
20	YEL029C_amp1	GAATGGAACACGATAGACGATTGGAGTC	AGTCAGATGCATGGGTACCTATTATGCC
21	YEL022W_amp3	CCACAGCGAGAAATCAACCAACGGTGCT	AGGATACAACTGTAATCTGTCGCCGCTG
22	YEL017W_amp1	CTCAACCGACAATGATAGCAGTACTAGC	GCTGCTGGTAGGCTGACCAGCA
23	YEL015W_amp2	AGTCTTGACCGTCGCTAGCCAAAGTGGT	GTCTTGTTGTGGCTGGCTTTGATGGGTA
24	YEL013W_amp1	TAGCTTGTTAAGCAGTACTGACCCAGAC	AACTAAGTGTGGTAAACCGCCGGCTCTA
25	YER010C_amp1	TCTACCGAAAACAACGGTACCGTTGCTC	CTCAAGTATCGTTGGTACCGCTTATACC
26	YEL011W_amp2	CTACGAAGCTCATGTTGGCATCAGCTCA	TAAACCGTCCTCAACATTCTTGCTGGCG
27	YER016W_amp1	CAGCAGCTTAGGTATCAATGGCTCACGT	GCCGTTAACCTCACCGTTTGAATTAGCG
28	YER020W_amp1	TCAACCTAGCTTAAGCGATGCCAGCTCA	GCTTGGTAAAGCCCATAAGGTGCTGATA
29	YER026C_amp1	TGATTTGCTGATCATACCGCAACCGTGA	CGGCAAGCCACACTATGTTCAGAGAGCT
30	YER027C_amp1	TGAGCTTGATGAGGCGTCAATGTCGTCG	CAACGAGGCCAGTTTAGCTTATACCTTC
31	YER028C_amp1	AGCCAAGCTTGAACCACTGCTGTGAACG	AGTCGCCCAGAACTGTGACGATGTTAAC
32	YER032W_amp1	ACGTAGCCCATTGAGATTTACCAGCAGC	CAATGGCTTAAAAGGCTCGATGCTAGGG
33	YER033C_amp3	GTTTTTACTACCACCTCTCAATGGGCTC	ACACAGCCAGCAGCCACATTACGCT
34	YER041W_amp2	CGGTCCTTCAAGCATCACCAGTCATTCA	ATGCATAATAGCAACGGTGCTAGGCCAA
35	YER042W_amp1	CGGTGAAGAGAGCAAGAAAGACTCACCA	GTGGGCAAATAAACCTGATCTGTACTGG
36	YER045C_amp1	ACTGTCACCGCTAGGAATGCTTGGG	CCCTTCAGCTGCTATCTATCCTTCATTC
37	YER047C_amp2	TGAACTTAAACTGCTCCACTGGACCAAG	TATGTTGTTGTTCGGTCCTCCTGGCACC
38	YER048C_amp1	ATCGTGTTTGACCATACCGCCGTCGGTA	CCACCCAGATAAACACCCAGACGATCCT

39	YER051W_amp1	CTCAGGTGTTTACGTTCCAAACGTTGGT	GTTCTCCTCAGCTTTCTCACCGTTAACC
40	YER053C_amp1	ACGCTTGCTAGCAACGCTCATACTCTCA	TAAGCAGCAGACCACCATGCCACCATTC
41	YER054C_amp2	GCTCTTGCTAGGAGGCATATCAGTTCTA	CAGATCAGGTAACGGCGTTCAAGCTCGT
42	YER055C_amp1	GCAGCTAGCCTCAACACTGCCTGAA	TTTCTTACCAGCCGCTGACATCCCTACC
43	YER056C_amp1	AAAAGCCAAGCCGGCGACCAAACTAAAG	CGCTTATGAGAAGTGGAGCTGGGTTCCA
44	YER059W_amp1	GATCTTGGATGGCGATACCTCAAATAGC	TGGGGTGGTGGTAACTGAGTTACAACGT
45	YER060C_amp2	TTTCAGCGTCTTTGGCGCTGAGTTAGGT	AGTGTAATCGGCAGCGTAGGTGGTCCAA
46	YER060C-A_amp2	CTTCTTCAGTGTTGTTGCTGGCTTGGCT	GGCAACGGTATACATACCTGGAACGTTG
47	YER061C_amp1	ACTACGACCAGGCAACAAAGCGCTAGCA	AACCGCTTGTGCTACCGGCAACAATAGT
48	YER062C_amp1	GTCTCTAGTGCCACTGGTAGCAACG	TGACGCCGAACATGTCATTCAAGTTAGC
49	YER064C_amp2	GTCAGCGCTGGTTGAGCTTCTGGTA	CGGTTTCGACGCTAGCTTAGCTCCTATT
50	YER065C_amp1	TCTGCTAAAATTGTGAACGGCCAAGGCA	TATGAGAGCTAGAGCTTTCGCCCCTTAC
51	YER066W_amp1	CCATCGTGCTTTGGTTGGCTTGTTAGGT	GGTGTCGCTTCTAACCAATAAACCTGAG
52	YER069W_amp1	CGATACCGCCAGCACTTTGAATAGCAGC	TGGCAAAGCCATGACCCAGAAATCAACG
53	YER070W_amp3	GCGTCCAGGCGCTTTCGCTTTGTATTTA	CTTCTCATATCTAGTGTACAAGGCCTCG
54	YER073W_amp2	CGGTGGTGCTCGTCACGGTTCA	GATGCCGCTCTGACCAAAACCGCCAAAT
55	YER075C_amp1	ACTGTCGCTTTCCAATGACAACATGCTC	TAGTGTCATCCCAGAATGGTTTCAGCAC
56	YER076C_amp1	GTCGCATGATGAATTGGTGCTCCAGGTT	CGGCGAAAGCACTTTGTGCCGTGCTAAA
57	YER081W_amp1	CGATTTGGATTATGCCACTTCACGTGGC	GTCTTTCATGGCGGCGAATTGAGGAGCT
58	YER086W_amp1	TAGCCAAGGCGTTGGCTTAAGCAGTAGA	TCTCTCTTCGGCTAACTTAGCGCATTCG
59	YER087W_amp1	TCAATGGCTACCTTTGGGTTTGCGTAGC	GTCAGCCCAAGCTGAGACGAATGGAATC
60	YER089C_amp1	ATCAGCGAAGCTTCTAACGCTGGTTCTA	CCCAGATATCTTGGAGCACAGCTTGGAC
61	YER091C_amp1	GATAGGAGGACGAACGTAACGACTGCCA	TCCTACCACCACCATCGGCAGTTTTCCA
62	YER096W_amp1	CAACCCTTCAGGTAGCGGTAGCAGCAAT	ATAAGCATCGCCCAACAAATACTGAGCG
63	YER098W_amp1	TGATCCAAGTATCGCTAAGAGCCCTTCA	AGCTCTGTCTTCGTAGGTGGTGCTACTG
64	YER099C_amp1	GACGTCGCCAACCAATAACATTCTGCTG	CGCTAACTTGTTAGAAACCGCTGGCTGC
65	YER101C_amp1	GCTATGTGATAAAATGTCGTAGCCGCCA	CGGCGCTGCTGTTTTAAGCGAACACTTT
66	YER103W_amp1	CGAAAGAGCTAAACGTACCTTAAGCAGC	GTAGGCGACAGCCTCGTCTGGATTGATA
67	YER105C_amp1	TGGAACTGAACCTGATTCACGGCTTGAC	CCCACAAGGCTATGCTAACGTTTTCGCT
68	YER107C_amp1	TGACAAGGCATAAGCGAAAACTGAGCCG	TAACCCAAACAGAGCTCCAGGTAGTAAC
69	YER109C_amp1	AGCGATTGACAAAGGACTGCTAGCGCTG	AGCCCCACCAACCAAGACCGCT
70	YER110C_amp1	ATCGTCAATATGACCGCTCAAGCTGCTA	TTGGAATGCTATCGACGAAAGTACCCGT
71	YER113C_amp2	ACCAACGCTGTTAGCCAATGACATCCAG	CCACTGCCCAGGTGCTAGCAAAAATTAT
72	YER114C_amp2	ACCACTGGCCAACAAAAATGGTGAACTG	CACCCCAACCGTCAGTTTATCAAAGGCT
73	YER115C_amp1	ATCGCATGAGAATCTGGCCAAAGGTGAA	TGCTTGCGATCCTAACAAACAGCAGAAC
74	YER116C_amp1	CTTTGAACGGCACAAGGCACAGTGACCA	CATGCACACTGAAGAGCCTGAAGCTTCA
75	YER118C_amp1	ACTCAACAAAATAACGCCAGCGCTGGCG	ATTTCCTAGATTCACCTGGTGGGGCATC
76	YER119C_amp1	AAAGCTGATGCTGGTACTACCGGTAGCA	CGCCACTGAATCAAGCCCATTAATCAGA
77	YER122C_amp1	ACGTGAGGCGGTCAAAATACTTGAACGG	AAGCGATAGCCCATTAGATACTGACAGC
78	YER129W_amp1	ATTGGCCGCCTCATCAACCAACTCAAGT	TGGAACATGATCGAAGTCCCAGCTGACG
79	YER130C_amp1	AACAACCAAACCGTGGCTGCTGCTACTT	CCAGACTGAAAATAGCAGTAGCCAGAAG

80	YER132C_amp1	TGATGAACGACGGCTTGGAGCCTTAGGA	CAGACGTGCTAGCCATCCATTGCAAAGC
81	YER141W_amp1	TAGACCTTTCAGCTTGAGTAGTCCAGTC	ACCAATGGCTCTGCCCCATAAACGG
82	YER143W_amp1	CAGCAGCGACAAACCATTGACCCCAACT	AAACAACAAACTAGCGGCGAATTCAGCG
83	YER144C_amp2	AGTGCTTGGGGTGTTCAACTTGCTCAAG	CAGCGTTATCAAGCCATTATCAGGTACC
84	YER151C_amp2	GCAACTGCTGGTACCAACACGGCTGTTA	CATCGAGCCTTTAGGCAGCATCGCTTTG
85	YER152C_amp1	ACTACGTAAGTGAGCGATACAACGCTGA	CTATAGCTTGGAGACTCGTAGACGTTTG
86	YER161C_amp1	TGGGCCGTTTGAATGCTTGCTCTTTGAA	AAGAAGCATCGGTGCTTCACACGCTCCT
87	YER163C_amp1	GCTGGTCAACAAAACTCTCTTGCCGCTC	AGCTAATCCTGGTCGTGTTGCTACCTTG
88	YER170W_amp1	TCGTTACGTCCATGTCCCATCAGGTCGT	GGTTTCACCGCTAACAGTGCCGAAGATA
89	YER174C_amp1	GTCGTCACTGCTACCTGATGATTCCTCG	CCCATGTAAGACCATGTCACAGGTTTTG
90	YER175C_amp1	CAATTCTGGTCTTCTTCTCAAGCTCTCC	CTGGGGCTACGCTGATCCTATCTTTCCA
91	YER177W_amp1	AAATGTCATCGGCGCCAGAAGAGCTAGC	GGTAGCTTTTTCACGGGCGTCACCTGAT
92	YER178W_amp1	TGCCAGCCGTAGTAGTGCTATGACC	ATCATAGGCCTTAACTTCGGCTTCGGTA
93	YER180C_amp1	TCTTTCAAAGACTGGGTAGGTACCGGCG	TTTGGATTACATCCCAGACAGTCCTAGC
94	YER182W_amp1	CGGTGGTTCATTCTTGGGTGGTTGGTAT	AACACCGCCAGGGGTGTTCAAAGCTCTA
95	YER184C_amp1	GTGTGAGGCGCTTGAGGCATCCAAGAAT	TGCTAGCTTGCAGAAGGGTTTGGCTAAT
96	YER188W_amp1	TCGTAGAAGCGACGCTTTGGGTGTTACC	GTTCCAACACAACATCTGACCAGGTCTG

References

1. Annaluru, N., Muller, H. & Mitchell, L. Total Synthesis of a Functional Designer Eukaryotic Chromosome. *Science* **344,** 55–58 (2014).
2. Xie, Z.-X. et al. 'Perfect' designer chromosome V and behavior of a ring derivative. *Science* **355,** (2017).
3. Wu, Y. *et al.* Bug mapping and fitness testing of chemically synthesized chromosome X. *Science* **355,** (2017).
4. Brachmann, C. B. *et al.* Designer deletion strains derived from Saccharomyces cerevisiae S288C: A useful set of strains and plasmids for PCR-mediated gene disruption and other applications. *Yeast* **14,** 115–132 (1998).
5. Cheng, T.-H. Controlling gene expression in yeast by inducible site-specific recombination. *Nucleic Acids Research* **28,** 108e–108 (2000).
